# Alterations of microbiota and metabolites in the feces of calves with diarrhea associated with rotavirus and coronavirus infections

**DOI:** 10.3389/fmicb.2023.1159637

**Published:** 2023-08-03

**Authors:** Shengwei Cui, Shihui Guo, Qingmei Zhao, Yong Li, Yun Ma, Yongtao Yu

**Affiliations:** ^1^School of Animal Science and Technology, Ningxia University, Yinchuan, China; ^2^College of Biological Science and Engineering, North Minzu University, Yinchuan, China; ^3^School of Life Sciences, Ningxia University, Yinchuan, China; ^4^Key Laboratory of Ruminant Molecular Cell Breeding in Ningxia, School of Animal Science and Technology, Ningxia University, Yinchuan, China

**Keywords:** calf diarrhea, intestinal microbiota, metabolites, bovine rotavirus, bovine coronavirus

## Abstract

The changes in the composition of intestinal microbiota and metabolites have been linked to digestive disorders in calves, especially neonatal calf diarrhea. Bovine rotavirus (BRV) and bovine coronavirus (BCoV) are known to be the primary culprits behind neonatal calf diarrhea. In this study, we analyzed changes in the fecal microbiota and metabolites of calves with neonatal diarrhea associated with BRV and BCoV infection using high-throughput 16S rRNA sequencing and metabolomics technology. The microbial diversity in the feces of calves infected with BRV and BCoV with diarrhea decreased significantly, and the composition changed significantly. The significant increase of *Fusobacterium* and the reductions of some bacteria genera, including *Faecalibacterium*, *Bifidobacterium*, *Ruminococcus*, *Subdoligranulum*, *Parabacteroides*, *Collinsella,* and *Olsenella*, etc., were closely related to diarrhea associated with BRV and BCoV infection. Metabolites in the feces of BRV and BCoV-infected calves with diarrhea were significantly changed. Phosphatidylcholine [PC; 16:1(9 Z)/16:1(9 Z)], lysophosphatidylethanolamine (LysoPE; 0:0/22:0), lysophosphatidylcholine (LysoPC; P-16:0) and LysoPE (0:0/18:0) were significantly higher in the feces of BRV-infected calves with diarrhea. In contrast, some others, such as desthiobiotin, were significantly lower. BRV infection affects glycerophospholipid metabolism and biotin metabolism in calves. Two differential metabolites were significantly increased, and 67 differential metabolites were significantly reduced in the feces of BCoV-infected calves with diarrhea. Seven significantly reduced metabolites, including deoxythymidylic acid (DTMP), dihydrobiopterin, dihydroneopterin triphosphate, cortexolone, cortisol, pantetheine, and pregnenolone sulfate, were enriched in the folate biosynthesis, pantothenate and CoA biosynthesis, pyrimidine metabolism, and steroid hormone biosynthesis pathway. The decrease in these metabolites was closely associated with increased harmful bacteria and reduced commensal bacteria. The content of short-chain fatty acids (SCFAs) such as acetic acid and propionic acid in the feces of BRV and BCoV-infected calves with diarrhea was lower than that of healthy calves, which was associated with the depletion of SCFAs-producing bacteria such as *Parabacteroides*, *Fournierella*, and *Collinsella*. The present study showed that BRV and BCoV infections changed the composition of the calf fecal microbiota and were associated with changes in fecal metabolites. This study lays the foundation for further revealing the roles of intestinal microbiota in neonatal calf diarrhea associated with BRV and BCoV infection.

## 1. Introduction

Diarrhea remains the most important cause of death in calves, especially during the first month after birth, which brings substantial economic losses to the dairy and beef cattle industries worldwide (Cho and Yoon, 2014; Aghakeshmiri et al., 2017). Enteric pathogen infection is the most crucial cause of neonatal calf diarrhea (Cho and Yoon, 2014). Bovine rotavirus (BRV), bovine coronavirus (BCoV), pathogenic *Escherichia coli*, and *Cryptosporidium* spp. are closely associated with neonatal calf diarrhea (Brunauer et al., 2021). Due to the complexity of diarrhea etiology, the lack of commercial vaccines, and the difference in management, neonatal calf diarrhea has not been controlled effectively in many countries. Intestinal microbiota plays an essential role in the formation and enhancement of the intestinal barrier, the regulation of immune function, and the maintenance of host health (Jandhyala et al., 2015). The disturbance of commensal microbiota in the gut has been associated with digestive disorders in neonatal animals. Characterizing the composition and function of the intestinal microbiota in diarrheal calves associated with pathogenic infections could provide insight into how to promote neonatal calf health by targeting the specific microbial community in the clinical treatment of diarrhea (Malmuthuge and Guan, 2017).

A growing number of investigations have shown that enteric pathogenic infections significantly alter the microbiota and metabolites in human and animal intestines, which is closely related to pathogen infection, intestinal epithelium inflammation, and digestive disorders (Mammeri et al., 2020; He L. et al., 2022). At present, there are few reports about the effects of BRV and BCoV infection on the intestinal microbiota of neonatal calves. A previous study based on a small cohort of animals indicates that BRV infection mediates the alteration of fecal microbiota in neonatal calves with diarrhea (Jang et al., 2019). The changes in the abundance of some bacteria genera are closely correlated with levels of physiological characteristics such as white blood cells, blood urea nitrogen, serum amyloid protein A, and glucose concentration in serum. Another study compared changes in fecal microbiota in post-weaned calves following recovery from BCoV-mediated diarrhea (Kwon et al., 2021). The changes in fecal microbiota in post-weaned calves with BCoV-associated diarrhea correlate with the changes of physiological parameters. In addition, diarrheal calves infected with *Cryptosporidium parvus*, another pathogen responsible for calf diarrhea, have a specific increase in the abundance of *Fusobacterium* in the fecal microbiota, which could be an important aggravating factor of cryptosporidiosis (Ichikawa-Seki et al., 2019). A recent study reveals that the infection of pathogenic *E. coli* causes significant changes in the colon microbiota of calves, with an increase in the abundance of harmful bacteria and a decrease in the abundance of symbiotic bacteria (He L. et al., 2022). Although the studies mentioned above confirm a strong association between changes in gut microbiota and enteropathogen infections closely associated with calf diarrhea, it is far from clear how changes in gut microbiota affect the physiology of neonatal calves and their exact role in the development of diarrhea associated with pathogenic microorganism infection. Moreover, BRV and BCoV are the major pathogens causing neonatal calf diarrhea. The effects of BRV and BCoV infection on the intestinal microbiota of neonatal calves are not defined well. Therefore, it is necessary to further characterize the response of gut microbiota to BRV and BCoV infection and the exact role of gut microbiota in the occurrence and development the pathogenic infection-associated diarrhea in neonatal calves, which is of great significance for the effective prevention and treatment of neonatal calf diarrhea in the future.

The intestinal metabolites derived from hosts and intestinal microbes, such as fatty acids, bacteria-transformed bile acids, amino acids, and vitamins, are pivotal in host energy metabolism and immune response regulation (Dobson et al., 2012; Kamada and Núñez, 2014; Chen and Stappenbeck, 2019; Van Treuren and Dodd, 2020). The disturbances of gut microbiota result in the alteration of gut metabolites, which adversely affect host metabolism and physiological homeostasis (Lin and Zhang, 2017; Dang and Marsland, 2019). The concentrations of some branch-chain fatty acids (BCFAs) are significantly increased in the feces of calves with diarrhea, which positively correlates with the changes in bacterial genera involved in lipid metabolism and butyrate synthesis (Xin et al., 2021). Some reports suggest that intestinal metabolites may influence infection and damage to the intestinal epithelium by enteropathogenic bacteria through different pathways (Grau et al., 2020; He Z. et al., 2022). For instance, *C. parvum* infection reduces the abundance of bacterial genera that contribute to the short-chain fatty acids (SCFAs) biosynthesis in the intestines of goat kids, particularly the butyrate biosynthesis, which could promote intestinal inflammation associated with *Cryptosporidium* infection (Mammeri et al., 2020). However, the characteristics of intestinal metabolites in diarrheal neonatal calves with BRV and BCoV infection, and their interaction with intestinal microbiota have yet to be fully understood. Previous studies mainly focus on the characteristics of intestinal microbiota composition in diarrheal neonatal calves associated with BRV infection without revealing the effect of the pathogenic infection on intestinal metabolic profile of neonatal calves, and the interaction of intestinal microbiota and intestinal metabolites in BRV and BCoV infection as well as the occurrence of diarrhea is not well investigated (Jang et al., 2019; Kwon et al., 2021). Consequently, it is essential to investigate the effects of BRV and BCoV infection on the intestinal metabolites of neonatal calves.

In the present study, we analyzed of microbiota and metabolites in the rectal feces samples from healthy neonatal calves with negative pathogen detection and calves with diarrhea positive for BRV or BCoV. The objectives were to characterize the profiles of microbiota and metabolites in feces samples from healthy calves without pathogenic infections and diarrheal calves associated with the infection of BRV or BCoV and further analyze the correlation among the microbiota, metabolites, and infection of BRV or BCoV. This will lay the foundation for further revealing the mechanism of intestinal microbiota and intestinal metabolites in the immune response of calves to major diarrhea-associated pathogens.

## 2. Materials and methods

### 2.1. Ethics statement

This study was approved by the Animal Experimental Ethical Review Committee of Ningxia University (Yinchuan, China; no. NXU-2019-051). The whole experiment process is in strict accordance with the National Standard Guidelines for the Ethical Review of Animal Welfare (GB/T 35892-2018).

### 2.2. Experimental animals and sample collection

The calves involved in this study were from a large-scale dairy farm in Ningxia Hui Autonomous Region, China. The calves were artificially fed with 4 L colostrum within 2 h of birth and then transferred to the calf island for separate feeding. Calves are fed 2 L of pasteurized milk each morning and evening during individual feeding on the calf island. Calves are given free access to water and food during feeding. All calves were female, and no antibiotics and probiotics were used before sampling.

Sample collection took place from June to September 2019. Calves with obvious diarrhea symptoms and healthy calves without any clinical symptoms within 30 days of age were selected as experimental subjects. The body temperature, respiration, and body condition of calves were examined before sampling. Fecal samples were collected from the rectum of calves using the rectum invasion method reported by Gomez et al. (2017). Feces were scored according to the standards of the Veterinary College of the University of Wisconsin-Madison (0 = normal feces, 1 = semi-formed feces, 2 = loose feces, 3 = watery feces; 0, 1 for health; 2,3 is diarrhea; McGuirk, 2008). The collected samples were subpackage in sterile feces collection tubes and cryotubes prepared in advance. The cryotubes were frozen in liquid nitrogen and returned to the laboratory for storage at −80°C. In the end, we collected 195 fecal samples, of which 89 were from healthy calves and 106 from diarrhoeic calves.

### 2.3. Sample detection and experimental grouping

**Figure 1:**
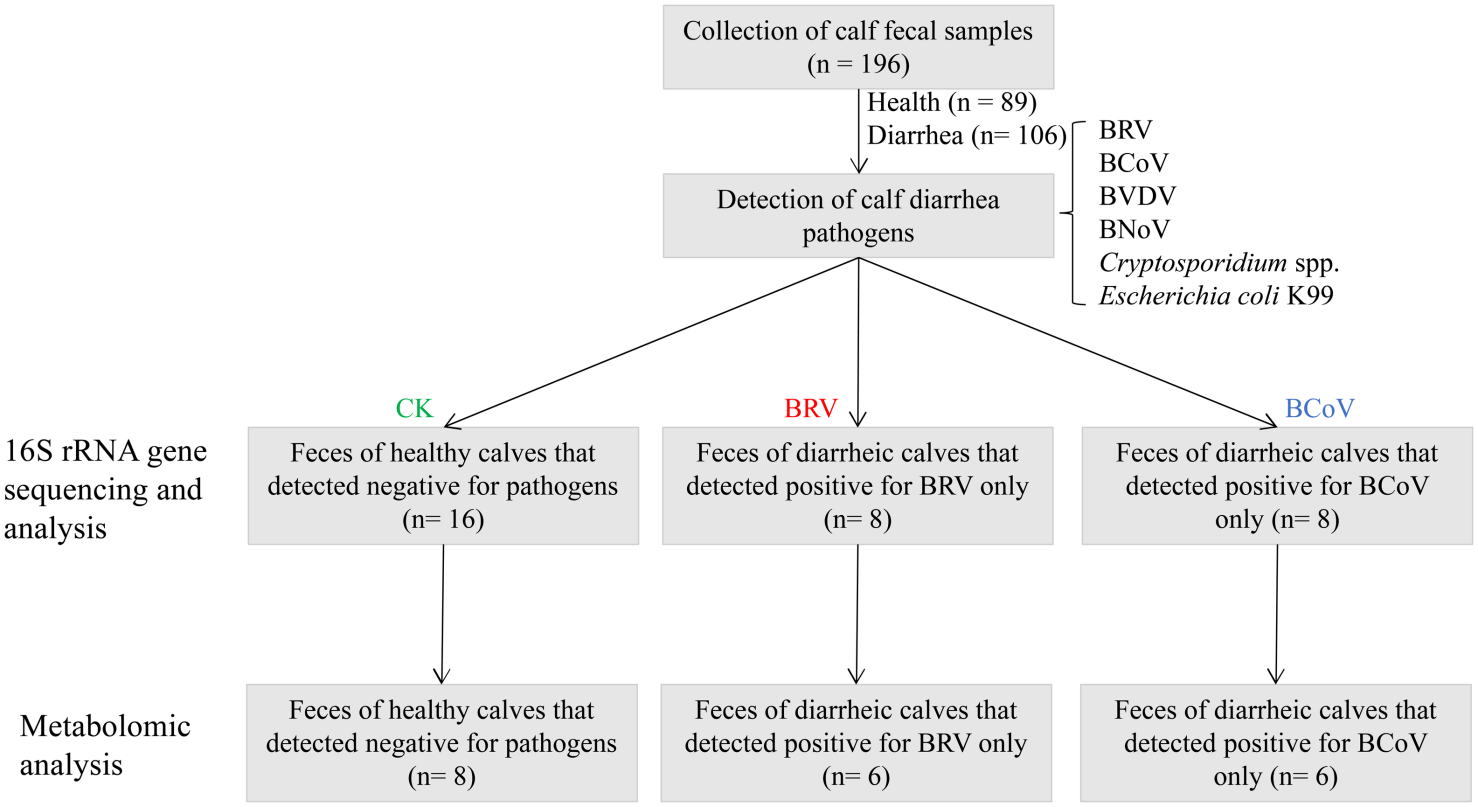
Flowchart of experimental design.

The collected samples were tested for major diarrhea-related pathogens, including BRV, BCoV, bovine viral diarrhea virus (BVDV), *Escherichia coli* K99, and *Cryptosporidium* spp. Firstly, antigen detection kits were used to detect BRV, BCoV, *Escherichia coli* K99, and BVDV in fecal samples (IDXX, United States). Subsequently, PCR was performed to detect BRV, BCoV, K99 *Escherichia coli*, BVDV, bovine norovirus (BNoV), and *Cryptosporidium* spp. in fecal samples (PCR primer information is shown in Supplementary Table 1. The pathogen detection results are shown in Supplementary Table 2). Based on the fecal score and pathogen detection results, samples with a fecal score of 0 and pathogen detection of negative samples were classified as group CK (*n* = 16). Samples with a fecal score of 3 and only detected BRV were classified as group BRV (*n* = 8), while samples with a fecal score of 3 and only detected BCoV were classified as group BCoV (*n* = 8). A total of 32 samples were used for 16S rRNA gene sequencing. Afterward, eight samples were selected from the CK group, six samples were selected from the BRV group, and six samples were selected from the BCoV group for metabolome sequencing analysis (Figure 1).

### 2.4. 16S rRNA gene sequencing

Genomic DNA was extracted from fecal samples (*n* = 32) using the E.Z.N.A. Stool DNA Kit (Omega Bio-Tek, Norcross, GA, United States). The quality and quantity of the DNA were assessed using NanoDrop 2000 ultraviolet–visible spectrophotometer (Thermo Fisher Scientific, Wilmington, NC, USA). DNA was diluted to 1 ng/μL using sterile double distilled water. The V3–V4 hypervariable region of the bacterial 16S rRNA genes was amplified by PCR using primers (341F 5′-CCTAYGGGRBGCASCAG-3′ and 806R 5′-GGACTACNNGGGTATCTAAT-3′; Qi et al., 2020). Thermal cycling consisted of initial denaturation at 98°C for 1 min, followed by 30 cycles of denaturation at 98°C for 10 s, annealing at 50°C for 30 s, and elongation at 72°C for 30 s. Finally, 72°C for 5 min. PCR products were mixed in equal concentrations according to the PCR products and their integrity was tested by electrophoresis using 2% agarose gel. The target fragments were purified using Qiagen Gel Extraction Kit (Qiagen, Hilden, Germany) for library construction. The 16S rRNA sequencing libraries were generated using TruSeq®DNA PCR-Free Sample Preparation Kit (Illumina, San Diego, CA, United States) following the manufacturer’s recommendations. The library quality was assessed on the Qubit@ 2.0 Fluorometer (Thermo Fisher Scientific, Wilmington, NC, United States) and Agilent Bioanalyzer 2,100 system (Agilent Technologies Inc. CA, United States). At last, the library was sequenced on an Illumina NovaSeq 6000 platform (Illumina, San Diego, CA, United States), and 250 bp paired-end reads were generated.

### 2.5. Illumina sequencing data analysis

The raw data of 16S rRNA gene sequencing were processed through a series of procedures, including data split, sequence assembly, filtration, and chimera removal to generate effective reads. OTUs clustering of effective data with 97% consistency was performed using Uparse software (Uparse Version 7.0.1001). The Silva Database was used based on the Mothur algorithm to annotate taxonomic information for each representative read. Multiple sequence alignments were performed using the MUSCLE software (Version 3.8.31) to analyze the phylogenetic relationship of different OTUs and the difference between the dominant species in different groups. The OTUs abundance information was normalized using the sequence numbering standard corresponding to the samples with the least sequences. Alpha diversity and beta diversity analysis were conducted based on normalized data using QIIME software (Version 1.9.1). Chao1 index, Shannon index, and Simpson index were used to the identify alpha diversity of community. PCoA was performed to evaluate the differences between groups in species complexity based on the Bray-Curtis distance. Based on Spearman’s correlation coefficient to correlation analysis of differential microbiota between groups. The Phylogenetic Investigation of Communities by Reconstruction of Unobserved States (PICRUSt, v2.5.0) was used to predict the microbial metabolic function based on the 16S rRNA gene sequencing data.

### 2.6. Analysis of untargeted metabolomics

A total of 20 samples were used for untargeted metabolomic assays, including 8 CK group samples, 6 BRV group samples, and 6 BCoV group samples. The 50 mg sample was transferred to a centrifuge tube with 400 μL methanol: water solution (4:1, v/v) and then treated by a frozen tissue grinder (Shanghai Wanbo Biotechnology Co., Ltd., Shanghai, China) at 50 Hz for 6 min. Samples were ground and then extracted in a low-temperature ultrasonic at 40 Hz for 30 min and then incubated at −20°C for 30 min. After incubation, the samples were centrifuged at 4°C 13000 g for 15 min, and the supernatants were transferred to sample vials for LC–MS/MS analysis. Ten microliters of supernatant were injected into a UHPLC system (Thermo Fisher Scientific Inc. MA, United States) equipped with BEH C18 (100 mm × 2.1 mm i.d., 1.8 μm; Waters, Milford, United States) and coupled with a Thermo Q Exactive Mass Spectrometer equipped with an electrospray interface for analysis of fecal metabolites. The mobile phases consisted of 0.1% formic acid (v/v) and the acetonitrile: isopropanol (1:1, v/v) containing 0.1% formic acid (v/v). A gradient elution procedure was performed for sample analysis. The flow rate was 0.4 mL/min, and the column temperature was set at 40°C. The mass spectrometric data was collected using a Thermo UHPLC-Q Exactive Mass Spectrometer equipped with an electrospray ionization (ESI) source operating in either positive or negative ion mode. The raw data were imported into the metabolomics processing software Progenesis QI (Waters Corporation, USA) for baseline filtering, peak identification, integration, retention time correction, and peak alignment. The relative abundance information of each metabolite ion in each sample was obtained after metabolite peak spectrum annotation, missing value filling, data normalization, QC sample RSD evaluation, and data conversion. Unsupervised principal component analysis (PCA) was performed to observe the overall distribution among the samples and the degree of dispersion among the different groups. Supervised Orthogonal Partial Least Squares Discrimination Analysis (OPLS-DA) was conducted to visualize differential metabolites among the different groups. KEGG pathway enrichment analysis was performed on the screened differential metabolites by Python software package scipy.stats.^1^ Based on Spearman’s correlation coefficient to correlation analysis of differential metabolites.

### 2.7. Analysis of short-chain fatty acids in fecal samples

The samples for SCFAs measurement were identical to samples for non-targeted metabolomics analysis. The 100 mg sample was weighed in a 2 mL grinding tube with a grinding bead and 450 μL methanol solution. The 2-ethyl butyric acid was also added as an internal standard to the grinding tube. Supernatants were obtained by grinding, extraction, and centrifugation of the sample in sequence according to the aforementioned methods. One microliter of supernatant was injected into an Agilent 8890B-5977B GC/MSD system (Agilent Technologies Inc. CA, United States) equipped with HP FFAP capillary column (30 m × 0.25 mm × 0.25 μm, Agilent J & W Scientific, Folsom, CA, United States) and electron bombardment ion source (EI) for GC/MS analysis. The split injection method was performed, and the split ratio was 10 to 1. Nitrogen was used as carrier gas at a flow ratio of 1.0 mL/min. The injection port temperature was 260°C. The column temperature was programmed at 80°C to 120°C at 40°C/min and from 120°C to 200°C at 10°C/min, and then held at 230°C for 1 min. The ion source temperature was 230°C. The quadrupole temperature was 150°C, the transfer line temperature was 230°C, and electron energy was set at 70 eV. The scanning mode was selected ion scanning mode (SIM). SCFAs were identified according to mass spectra and retention time of corresponding standards (Sigma Aldrich, St. Louis, MO, United States). The linear regression standard curves were done according to the concentrations of SCFAs standard in aliquots and the ratio of SCFAs peak area and internal standard peak area. The SCFA concentration of each sample was calculated from the standard curve. Based on the relative abundance of fecal microbiota and Spearman’s correlation coefficient of SCFAs content in feces to evaluate the correlation between fecal microbiota and SCFAs.

### 2.8. Statistical analysis

An analysis of alpha diversity indices (Chao1 index, Shannon index, and Simpson index) was performed to compare the different groups based on the Wilcox rank sum test. Permutational multivariate analysis of variance (PERMANOVA) was used to analyze differences in fecal microbiota structure between groups. A Wilcoxon test was used to analyze the difference in bacterial microbiota between groups. *T*-test combined with the multivariate analysis OPLS-DA method was used to screen out the differential metabolites between groups. A 200-time permutation test was used to examine the fitting effect of the model. The SCFAs content in the two groups of samples was compared using the Kruskal-Wallis test in Origin 2021 (Version 9.8.0.200) and plotted using GraphPad Prism 8.0.1 (Version 8.0.1.244). The relationships between microbiota and SCFAs were analyzed using Spearman’s correlation test. All values were shown as mean ± standard error of the mean, and *p* < 0.05 was considered a significant difference.

## 3. Results

### 3.1. Alterations of microbiota in the feces of calves with diarrhea associated with rotavirus and coronavirus infections

A total of 2,042,707 valid reads were obtained from 32 fecal samples, with an average of 63,834 reads per sample (Supplementary Table 3). The sample dilution curve indicated that the sequencing depth was reasonable and the sequencing data could reflect most of the microbial information (Supplementary Figure 1A). A total of 3,032 OTUs were obtained with cluster analysis (Supplementary Figure 1B). There were 928 OTUs in the CK group, 1923 OTUs in the BRV group, and 2,563 OTUs in the BCoV group.

**Figure 2:**
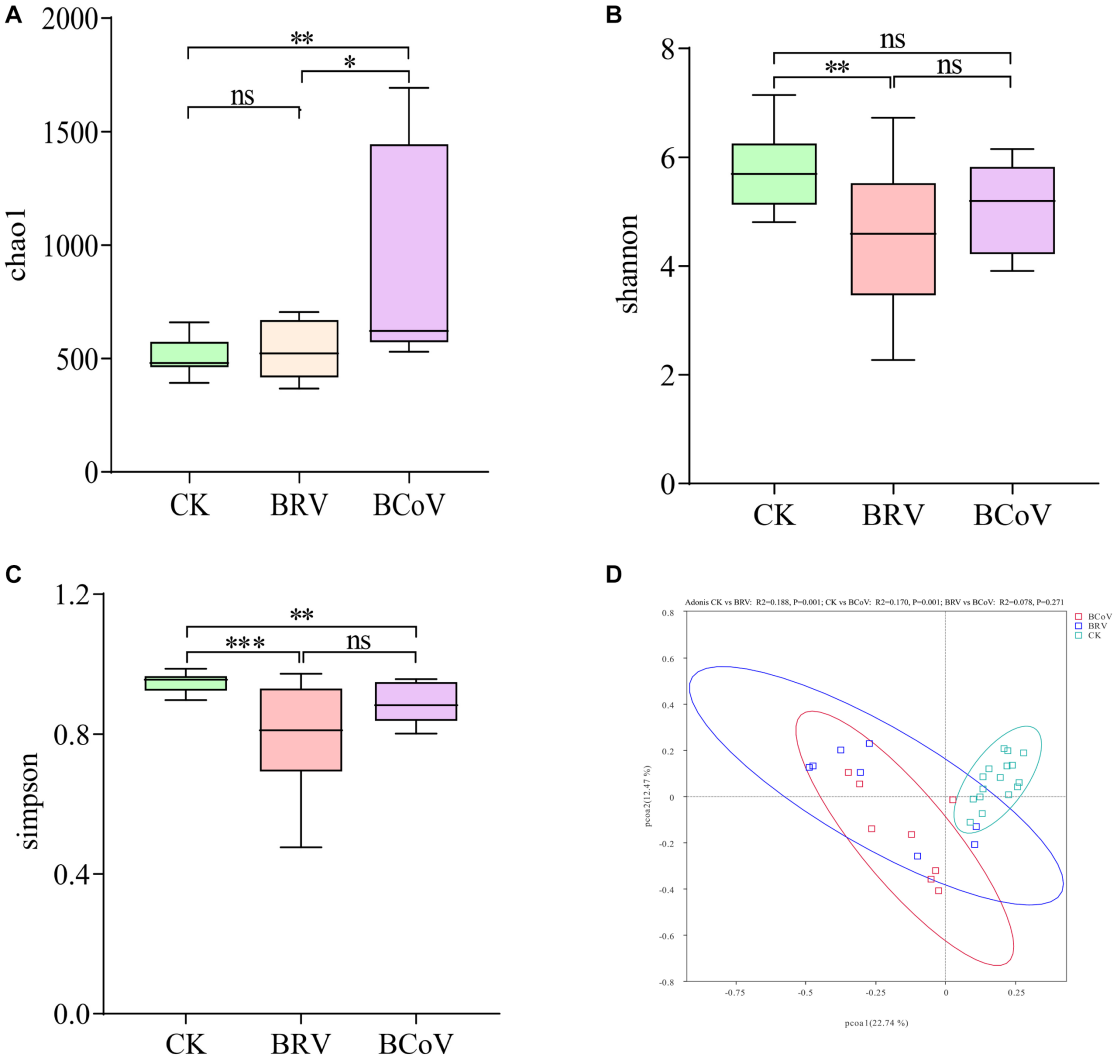
Calf fecal microbiota diversity. **(A)** Chao 1 index box plot. **(B)** Shannon index box plot. **(C)** Simpson index box plot. **(D)** PCoA diagram. The top and bottom borders of the box plot represent the maximum and minimum values, and the middle line represents the median. ^*^p < 0.05, ^**^p < 0.01, ^***^p < 0.001, ns*P* > 0.05.

The results of alpha diversity analysis showed that the Chao 1 indices of the BRV group were not significantly different from those of the CK group (Figure 2A). While the BRV group had extremely significantly lower Shannon indices and Simpson indices than the CK group (*p* < 0.01; Figures 2B,C). Compared with the CK group, Chao 1 indices of the BCoV group were extremely significantly increased (*p* < 0.01; Figure 2A), and Simpson indices were extremely significantly decreased (*p* < 0.01; Figure 2C). However, no significant difference between the CK group and the BCoV groups was observed in Shannon indices (Figure 2B). There was no significant difference in Shannon indices and Simpson indices between the BRV group and the BCoV group (Figures 2B,C). However, the Chao 1 index was significantly higher in the BCoV group than in the BRV group (Figure 2A). PCoA plots based on Bray-Curtis distances that the CK group differed from the BRV and BCoV group samples (Figure 2D). Based on the PERMANOVA results, the fecal microbiota of the CK group was significantly different from that of the BRV and BCoV groups. Still, the difference between the BRV and BCoV group’s fecal microbiota was insignificant.

For the microbiota composition, at the phylum level, the dominant bacterial phyla in the CK group were Firmicutes, Bacteroidota, Actinobacteria, and Proteobacteria (Supplementary Figures 2A,B). Compared with the CK group, the abundance of Fusobacteriota extremely significantly increased in the BRV group (*p* < 0.001). In contrast, the abundance of Actinobacteria and Euryarchaeota extremely significantly decreased (*p* < 0.001; Supplementary Figure 2C). The abundance of Fusobacteriota, Acidobacteria, and Proteobacteria extremely significantly increased (*p* < 0.01), while the abundance of Actinobacteria extremely significantly decreased (*p* < 0.01), and the abundance of Euryarchaeota significantly decreased in the BCoV group (*p* < 0.05; Supplementary Figure 2D).

**Figure 3:**
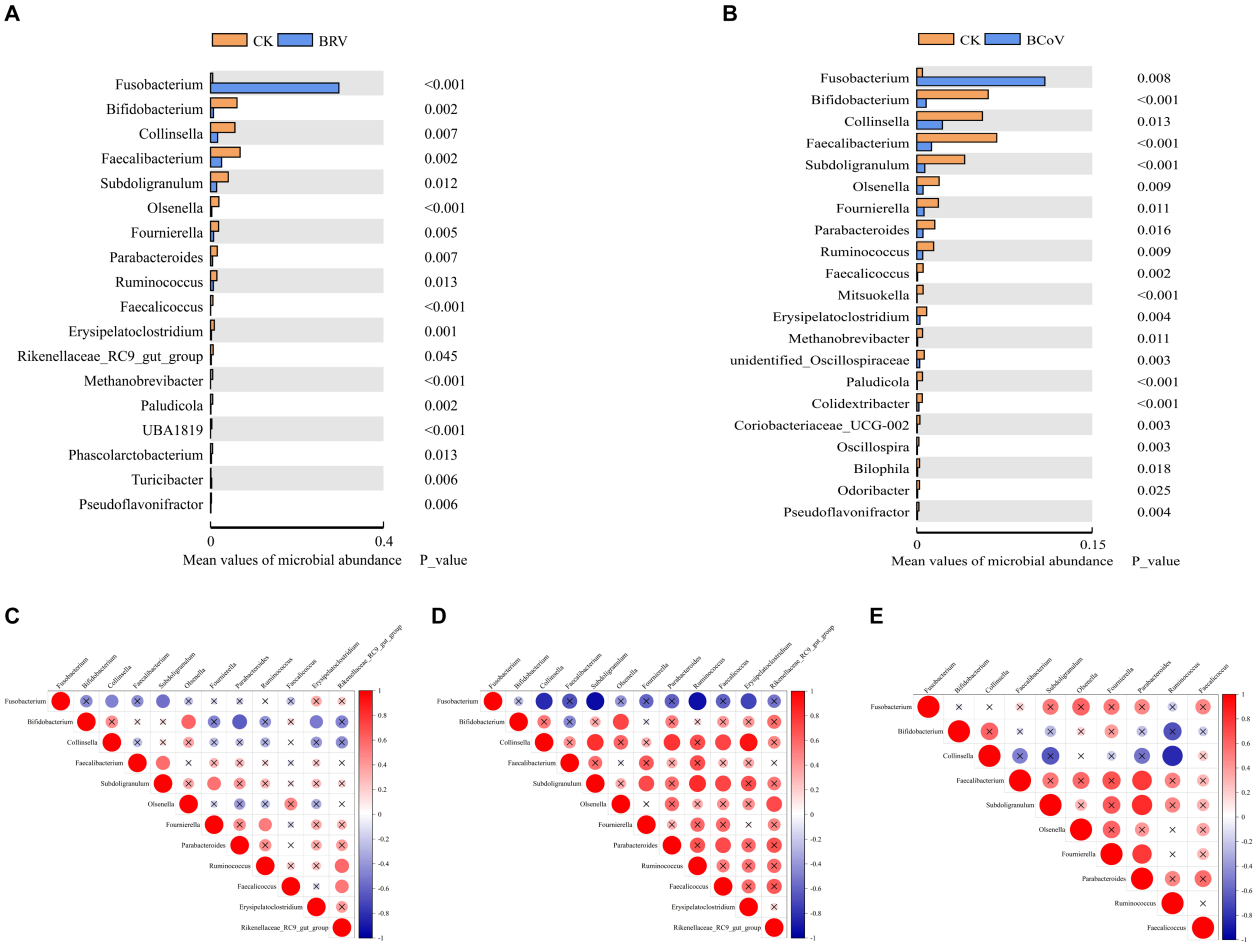
Differential microbiota between groups and correlation analysis. **(A)**Wilcoxon tests for genus levels in the CK and BRV groups. **(B)**Wilcoxon tests for genus levels in the CK and BCoV groups. **(C)** Correlation between different microbiota in the CK group. **(D)** Correlation between different microbiota in the BRV group. **(E)** Correlation between different microbiota in the BCoV group. In the correlation map, positive correlations are shown in red, negative correlations in blue, and the size of the circles indicates the correlation coefficient, with larger circles indicating larger values. A *p-*value > 0.05 is considered insignificant. A cross is added to the circle indicating the correlation coefficient in this case.

At the genus level, The dominant bacterial genera in the CK group were *Bacteroides*, *Faecalibacterium*, *Bifidobacterium*, etc. (Supplementary Figures 2E,F). Compared with the CK group, the relative abundance of *Faecalibacterium*, *Bifidobacterium*, *Collinsella*, *Fournierella*, *Erysipelatoclostridium*, *Parabacteroides*, *Olsenella*, and *Faecalicoccus*, etc., extremely significantly decreased (*p* < 0.01), and the relative abundance of *Subdoligranulum*, *Ruminococcus*, *Rikenellaceae_RC9_ gut_group*, etc., significantly decreased in the BRV group (*p* < 0.05; Figure 3A). The relative abundances of *Bifidobacterium*, *Faecalibacterium*, *Subdoligranulum*, *Olsenella*, and *Ruminococcus*, etc., were extremely significantly decreased (*p* < 0.01), and the relative abundances of *Collinsella*, *Fournierella*, and *Parabacteroides*, etc., were decreased in BCoV group (*p* < 0.05; Figure 3B). The BRV and BCoV groups had an extremely significantly higher abundance of *Fusobacterium* than the CK group (*p* < 0.01).

Microbiota correlation analysis showed that *Fusobacterium* was negatively correlated with *Bifidobacterium*, *Faecalibacterium*, and *Parabacteroides* in the CK group and significantly negatively correlated with *Collinsella*, *Subdoligarnulum*. *Bifidobacterium* was significantly positively correlated with *Olsenella* and negatively correlated with *Parabacteroides* and *Erysipelatoclostridium*. *Subdoligarnulum* was significantly positively correlated with *Faecalibacterium* and *Fournierella*. *Subdoligarnulum* showed a significant positive correlation with *Faecalibacterium* and *Fournierella* (Figure 3C). In the BRV group, *Fusobacterium* was negatively correlated with *Bifidobacterium*, *Faecalibacterium*, *Parabacteroides*, etc., and significantly negatively correlated with *Collinsella*, *Subdoligarnulum*, *Ruminococcus*, and *Erysipelatoclostridium*, etc. *Bifidobacterium* was positively correlated with *Parabacteroides*, *Erysipelatoclostridium*, etc., and significantly negatively correlated with *Olsenella*, but negatively correlated with *Faecalibacterium*. *Parabacteroides*, *Ruminococcus*, *Faecalicoccus*, etc. also showed positive or significant positive correlations with each other (Figure 3D). In the BCoV group, *Fusobacterium* was negatively correlated with *Bifidobacterium*, *Collinsella*, etc., and positively correlated with *Faecalibacterium*, *Parabacteroides*, etc. *Faecalibacterium*, *Faecalibacterium*, *Fournierella* was significantly positively correlated with *Parabacteroides*, and *Collinsella* was significantly negatively correlated with *Ruminococcus* (Figure 3E).

**Figure 4:**
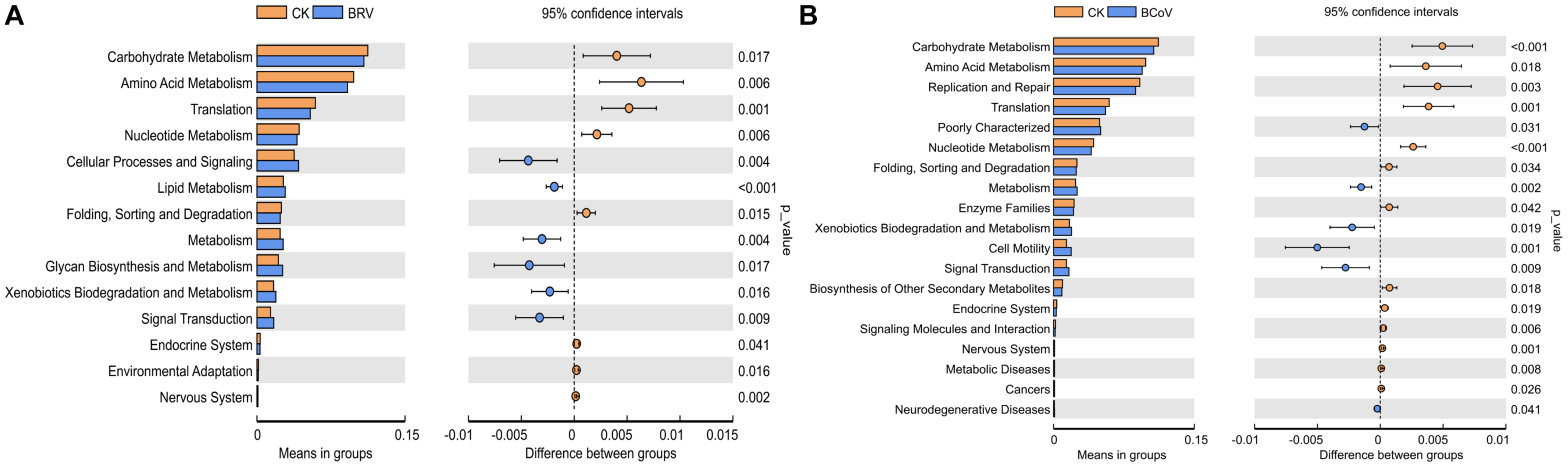
Functional changes in the intestinal microbiota of calves. **(A)** Differences in metabolic pathways between the CK and BRV groups. **(B)** Differences in metabolic pathways between the CK and BCoV groups.

Based on PICRUSt functional prediction analysis, 39 pathways were detected in the CK and BRV groups, of which 17 pathways were significantly different in abundance in the CK and BRV groups (Figure 4A). Compared to the CK group, the BRV group showed a significantly higher abundance of lipid metabolism, glycan biosynthesis and metabolism, signaling transduction, immune system diseases, and excretory system, and a significantly lower abundance of carbohydrate metabolism, amino acid metabolism, nucleotide metabolism, and digestive system, etc. In the CK and BCoV groups, 41 pathways were detected, 22 of which were significantly different in abundance in the CK and BCoV groups (Figure 4B). Compared to the CK group, the BCoV group showed a significantly higher abundance of xenobiotics biodegradation and metabolism, biosynthesis of other secondary metabolites, signaling transduction, and immune system diseases, and a significantly lower abundance of carbohydrate metabolism, amino acid metabolism, nucleotide metabolism, enzyme families, and immune system, etc.

### 3.2. Alterations of metabolites in the feces of calves with diarrhea associated with rotavirus and coronavirus infections

A total of 1,626 metabolites were obtained after quality control. PCA plots in positive and negative ion modes showed that the BRV group was completely separated from the CK group (Supplementary Figures 3A,B). BCoV group was also wholly separated from the CK group (Supplementary Figures 3C,D). OPLS-DA results demonstrated that the Q2 and Y axis intercepts of the BRV group and BCoV group vs. the CK group were less than 0.05 under positive and negative ion modes (Supplementary Figures 3E–L). The models were stable and had good interpretability and predictability.

**Figure 5:**
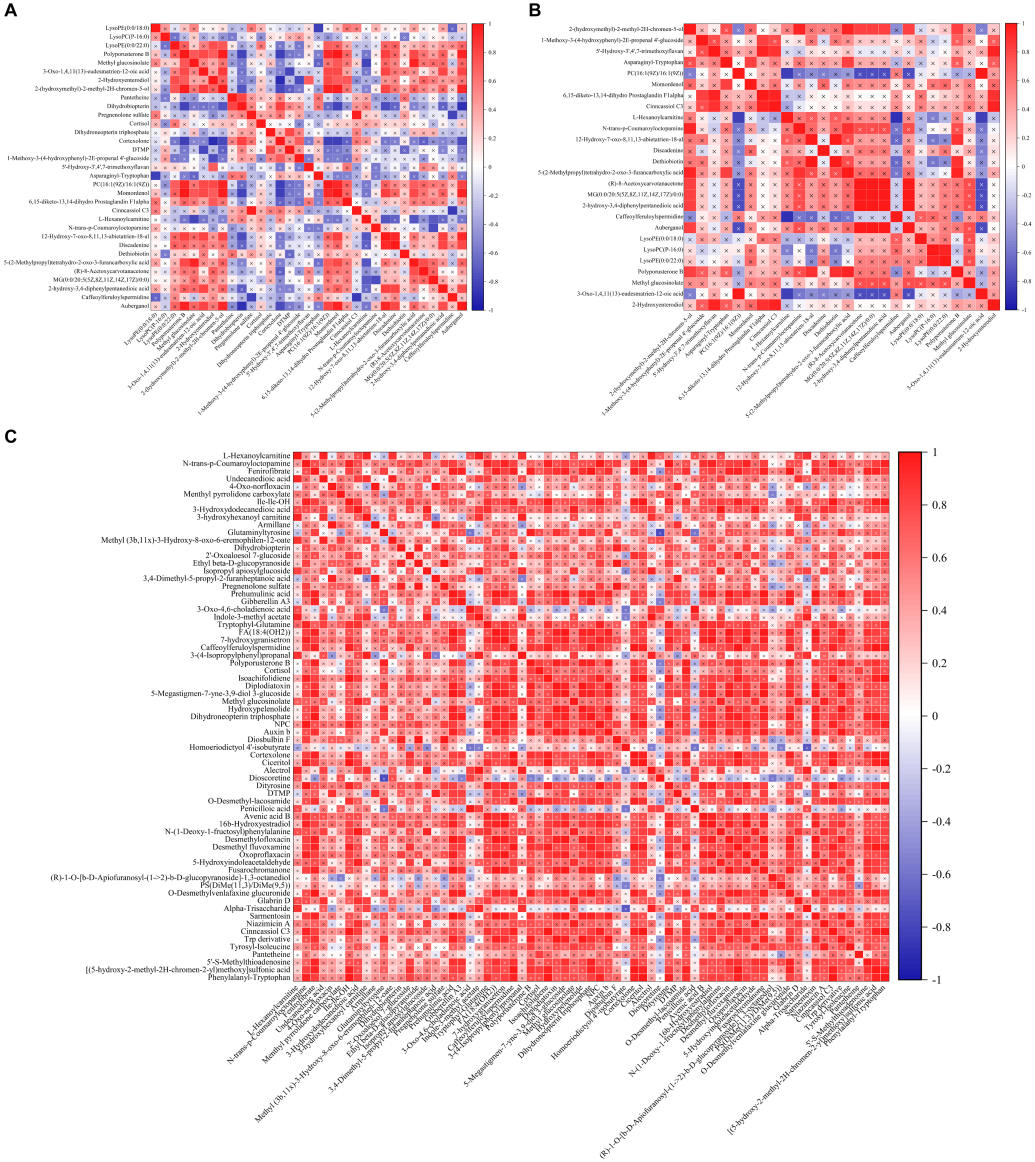
Correlation analysis of differential metabolites in feces. **(A)** Correlation between different metabolites in the CK group. **(B)** Correlation between different metabolites in the BRV group. **(C)** Correlation between different metabolites in the BCoV group. In the correlation map, the color indicates the correlation coefficient. Positive correlations are shown in red and negative correlations in blue. A cross has been added to the box to indicate a value of *p* of >0.05 for the correlation coefficient. Otherwise, all indicate a significant correlation.

The threshold values were *p* < 0.05, VIP > 2, FC > 1, or < −1 to screen differential metabolites. There were 26 differential metabolites between the CK group and BRV group in positive and negative ion modes (Supplementary Table 4), among which phosphatidylcholine [PC; 16:1(9 Z)/16:1(9 Z)], lysophosphatidylethanolamine (LysoPE; 0:0/22:0), lysophosphatidylcholine (LysoPC; P-16:0), and LysoPE (0:0/18:0) were upregulated in BRV group. However, 22 metabolites, including dethiobiotin, were downregulated in the BRV group. There were 69 differential metabolites between the CK and BCoV groups under positive and negative ion modes (Supplementary Table 5). Among them, (R)-1-O-[b-D-Apiofuranosyl-(1 → 2)-b-D-glucopyranoside]-1, 3-octanediol and phosphatidylserine [PS; DiMe (11, 3)/DiMe (9, 5)] were upregulated in BCoV group, and 67 differential metabolites were downregulated. Correlation analysis of differential metabolites between groups showed a significant correlation between metabolites (Figure 5). For example, in the CK group, PC [16:1(9 Z)/16:1(9 Z)] was significantly negatively correlated with cortexolone and significantly positively correlated with LysoPE (0:0/22:0), polyporusterone B, and momordenol, etc. Dihydrobiopterin was significantly negatively correlated with polyporusterone B, momordenol, etc., and significantly positively correlated with pantetheine and cortexolone (Figure 5A). In the BRV group, L-Hexanoylcarnitine was significantly negatively correlated with PC [16:1(9 Z)/16:1(9 Z)], Caffeoylferuloylspermidine. Dethiobiotin and LysoPE (0:0/22:0) were significantly and positively correlated with polyporusterone B and LysoPC (P-16:0), respectively (Figure 5B). In the BCoV group, a positive or significant positive correlation was observed between various metabolites such as DTMP, cortexolone, pantetheine, and dihydrobiopterin (Figure 5C).

The differential metabolites from the BRV group and BCoV group were performed enrichment analysis of the KEGG pathway. The impact-value threshold was set to 0.05 to identify the most relevant metabolic pathways. Two differential metabolites of the BRV group, PC [16:1(9 Z)/16:1(9 Z)] and dethiobiotin, were enriched in glycerophospholipid metabolism and biotin metabolism pathways, respectively. In the BCoV group, there were seven differential metabolites that, including deoxythymidylic acid (DTMP), dihydrobiopterin, dihydroneopterin triphosphate, cortexolone, cortisol, pantetheine, and pregnenolone sulfate were enriched, respectively, in the pathways of folate biosynthesis, pantothenate and CoA biosynthesis, pyrimidine metabolism, and steroid hormone biosynthesis.

### 3.3. Alterations of SCFAs in the feces of calves with diarrhea associated with rotavirus and coronavirus infection

Rotavirus and coronavirus infections differentially affect SCFAs content in calf feces. Compared with the CK group, the BRV group had significantly lower contents of total SCFAs, acetic acid, propanoic acid, and isohexanoic acid (*p* < 0.05; Supplementary Figure 4A). However, only the content of propanoic acid in the BCoV group decreased significantly (*p* < 0.05; Supplementary Figure 4B).

### 3.4. Correlation of microbiota and metabolites in feces

**Figure 6:**
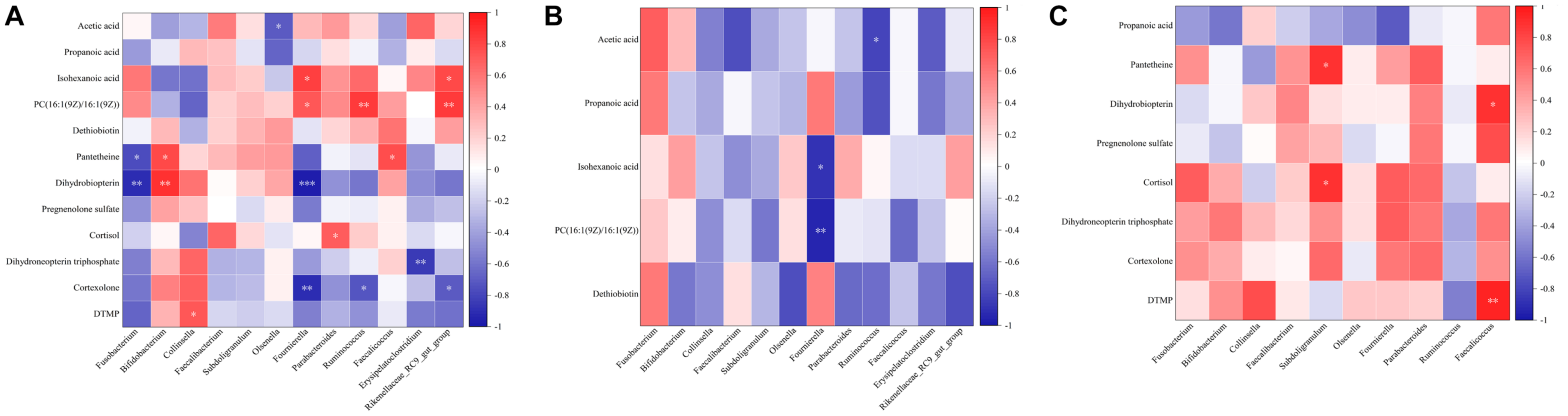
Correlation analysis of microbiota and metabolites in feces. **(A)** Correlation analysis of differential microbiota and metabolites in the CK group. **(B)** Correlation analysis of differential microbiota and metabolites in the BRV group. **(C)** Correlation analysis of differential microbiota and metabolites in the BCoV group. In the correlation map, the color indicates the correlation coefficient. The horizontal coordinate is the microbiota, and the vertical coordinate is the metabolites. Positive correlations shown in red and negative correlations in blue. The abscissa coordinates are microorganisms, and the ordinate coordinates are metabolites. ^*^p < 0.05, ^**^p < 0.01, ^***^p < 0.001.

To understand the correlation between microbiota and metabolites in feces, we focused on the relationship between significantly different microbiota and metabolites (Figure 6). In the CK group, PC [16:1(9 Z)/16:1(9 Z)] was negatively correlated with *Bifidobacterium* and *Collinsella*, positively correlated with *Fusobacterium*, and significantly correlated with *Fournierella*, *Ruminococcus*, *Rikenellaceae_RC9_gut_ group*. Pantetheine was significantly and negatively correlated with *Fusobacterium* and positively correlated with *Bifidobacterium* and *Faecalicoccus*. Dihydrobiopterin was significantly and negatively correlated with *Fusobacterium* and *Fournierella* and positively correlated with *Bifidobacterium* was significantly positively correlated (Figure 6A). In the BRV group, PC [16:1(9 Z)/16:1(9 Z)] was significantly negatively correlated with *Fournierella* and positively correlated with *Fusobacterium*, *Bifidobacterium*, etc. Acetic acid was significantly negatively correlated with *Ruminococcus* and positively correlated with *Bifidobacterium* isohexanoic acid was significantly negatively correlated with *Fournierella* and positively correlated with *Bifidobacterium*, *Olsenella*, *Parabacteroides*, *Rikenellaceae_RC9_gut_group*, etc. Dethiobiotin was negatively correlated with *Faecalibacterium*, *Fournierella*, and others (Figure 6B). In the BCoV group, propanoic acid was negatively correlated with *Fusobacterium* and *Fournierella* and positively correlated with *Collinsella* and *Faecalicoccus*. DTMP, cortexolone, dihydroneopterin triphosphate, dihydrobiopterin, pregnenolone sulfate, pantetheine, and cortisol were positively or significantly correlated with *Faecalibacterium*, *Subdoligranulum*, *Parabacteroides*, *Faecalicoccus*, etc. (Figure 6C).

## 4. Discussion

In the present study, diarrheal calves with BRV or BCoV infection had a relatively higher quantity of OUTs and higher Chao 1 indices than healthy calves, suggesting that BRV or BCoV infection increased the number of bacterial species in calf feces. In addition, Shannon indices and Simpson indices of fecal microbiota decreased significantly in the diarrhea group associated with BRV infection, whereas only Simpson indices were reduced significantly in the diarrhea group associated with BCoV infection, indicating that both BRV and BCoV infection reduced the diversity of the gut microbiota in neonatal calves. In a previous study, Chao 1 indices and Shannon indices were significantly reduced in the feces of diarrheal calves with BRV infection, while Simpson indices were significantly increased (Jang et al., 2019). Similarly, significant reductions in the α diversity of gut microbiota have been observed in human infants and other studied species (Engevik et al., 2020; Xiong et al., 2021; Zhao et al., 2021). There are some agreements or contradictions between our results and previous studies. Only Chao 1 indices significantly increased in the fecal microbiota of human infants infected with rotavirus, while no significant difference is observed in all alpha diversity indices of the fecal microbiota of suckling mice with rotavirus-associated diarrhea (Xiong et al., 2021; Zhao et al., 2021). An explanation for these differences would be that changes in the gut microbiota of newborn animals are influenced by a variety of factors, including environment, diet, age, gender, and the type and virulence of viruses infected (Castro et al., 2016). Although the effects of BCoV infection on the intestinal microbiota of neonatal calves have not been documented in previous studies, a study in piglets showed that coronavirus infection can significantly reduce the diversity of intestinal microbiota (Li et al., 2020).

Previous studies have shown that diarrheal calves develop dysbiosis of intestinal microbiota, with the structure and membership of their intestinal microbiota significantly different from that of healthy calves (Gomez et al., 2017). Diarrheal calves have a higher abundance of opportunistic pathogens, especially members of Proteobacteria, Fusobacteriota which is often associated with intestinal dysbiosis (Zeineldin et al., 2018; Fan et al., 2021). Further research has shown that the changes in gut microbiota may be related to the replication and pathogenesis of virus infection (Gomaa, 2020; Xiong et al., 2021). Jang et al. found that the increase of relative abundance in genera *Escherichia* and *Clostridium* were was closely related to BRV infection in neonatal calves (Jang et al., 2019). *Enterococcus* and *Escherichia-Shigella* genera were significantly enriched in the rectal feces of newborn mice infected with rhesus rotavirus (Zhao et al., 2021). Sohail et al. suggest a significant increase in gut microbiome diversity denoted by higher relative abundances of phylum Proteobacteria, Fusobacteria, and genus *Streptococcus* in children with rotavirus infection (Sohail et al., 2021). The effects of BCoV infection on the intestinal microbiota of neonatal calves have not been reported. However, some studies in humans and other animal species show that there is a close association between coronavirus infection and significant changes in intestinal microbiota composition. For instance, the depletion of *Bifidobacterium* and *Faecalibacterium* in the gut is associated with SARS-CoV-2 infection severity (Hazan et al., 2022). Another study has demonstrated that diarrheal piglets infected with porcine delta coronavirus (PDCoV) infection have a significantly lower abundance of Bacteroidetes and a significantly higher abundance of Firmicutes in the colon and feces of piglets (Li et al., 2020). In the current investigation, diarrheal calves infected with BRV and BCoV had similar changes in the abundance and composition of fecal microbiota. In two diarrhea groups, the abundance of Fusobacteriota extremely significantly increased, and Actinobacteria and Euryarchaeota were extremely significantly reduced. At the genus level, the significant increase of *Fusobacterium* and the deletions of several bacteria genera, including *Faecalibacterium*, *Bifidobacterium*, *Ruminococcus*, *Subdoligranulum*, *Faecalicoccus*, *Parabacteroides*, *Fournierella*, *Collinsella*, and *Olsenella* were observed in both two diarrheal groups. The differences were the excessive presence of Proteobacteria in the BCoV group as well as the loss of *Erysipelatoclostridium*, and *Rikenellaceae_RC9_gut_group* in the BRV group. The aforementioned studies have not found a strong association between rotavirus or coronavirus infection and a significant increase in the abundance of *Fusobacterium* in the gut. The reasons accounting for these differences are unclear, but differences in methodologies, management, pathogen, and the host could be responsible for the differences in intestinal microbiota. *Fusobacterium* is usually commensal bacteria in the oral cavity and gastrointestinal tract associated with human Crohn’s disease, ulcerative colitis and colorectal cancer (Zhou et al., 2018; Brennan and Garrett, 2019). It has been reported that the increase of *Fusobacterium* could promote the development of ulcerative colitis and modulate gut epithelial cell innate immunity (Martin-Gallausiaux et al., 2020; Su et al., 2020). It has also been shown that *Cryptosporidium* infection can specifically increase the abundance of *Fusobacterium* in the intestinal tract of neonatal calves. The excessive presence of this bacteria genera in the intestinal tract would aggravate the symptoms of diarrhea caused by *Cryptosporidium* (Ichikawa-Seki et al., 2019).

The results based on PICRUSt functional predictions show that virus-infected diarrhoeic calves also show significant changes in microbiota function compared to healthy calves. The viral infection affects multiple pathways of carbohydrate metabolism, amino acid metabolism, lipid metabolism, nucleotide metabolism, the immune system, and the digestive system. Firmicutes and Bacteroidota are involved in the degradation of complex polysaccharides that help calves metabolize carbohydrates, sugars, and fatty acids, and a reduction in their abundance can lead to a decline in their function (Jami et al., 2013). At the same time, short-chain fatty acids play an essential role in maintaining metabolism and immunity in the body, and the absence of short-chain fatty acid-producing microbiota can reduce immune function (Yang et al., 2020). The above evidence indicated that the infection of BRV and BCoV caused dysbiosis of intestinal microbiota in neonatal calves and significantly changed the intestinal microbiota’s structure, composition, and function. However, it remains to be further studied whether the characteristics of the intestinal microbiota are prevalent in neonatal diarrheal calves infected with BRV or BCoV under different feeding conditions and management modes.

In this study, compared with the healthy group, calves with diarrhea associated with BRV or BCoV infection had significantly changed in fecal metabolites. Four upregulated differential metabolites, such as PC [16:1(9 Z)/16:1(9 Z)], LysoPE (0:0/22:0), LysoPC (P-16:0), and LysoPE (0:0/18:0) were increased significantly in the BRV infection-associated diarrhea group. Among them, PC [16:1 (9 Z)/16:1(9 Z)] was enriched in the Glycerophospholipid metabolism pathways. PC [16:1(9 Z)/16:1(9 Z)] is phosphatidylcholine (PE), while LysoPE and LysoPC belong to the lysophospholipid class of compounds. These compounds are also considered to be important components of the lipid droplet monolayers in mammalian cells. It has been reported that rotavirus induces the formation of lipid droplets in infected cells by upregulating the synthesis of lipid molecules, which may promote viral replication in host cells (Gaunt et al., 2013; Criglar et al., 2022). In addition, dethiobiotin, as a major precursor of biotin, was significantly downregulated in the feces of BRV-infected diarrheal calves and was enriched in the Biotin metabolic pathway in this study. Biotin is a cofactor of carboxylases, which is produced by different species of gut bacteria and is essential for glucose, amino acid, and fatty acid metabolism (Bi et al., 2016; Yoshii et al., 2019). It can exert anti-inflammatory effects by inhibiting the activity of NF-κB (Elahi et al., 2018). There is some evidence that free biotin is essential for the growth and survival of the microbiota, and biotin deficiency affects the composition of the intestinal microbiota, leading to gut dysbiosis (Hayashi et al., 2017). In our study, a strong correlation was observed between the excessive presence of *Fusobacterium* and the loss of some potentially beneficial bacteria genera (Yoshii et al., 2019).

In the BCoV group, PS [DiMe (11, 3)/DiMe (9, 5)] and (R)-1-O-[b-D-Apiofuranosyl-(1 → 2)-b-D-glucopyranoside]-1, 3-octanediol were upregulated in the present study. PS [DiMe(9, 5)/DiMe(11, 3)] belongs to the phosphatidylserine class of organic compounds (PS; Djoumbou Feunang et al., 2016). In mammalian cells, PS is mainly distributed in the inner monolayer surface of the plasma membrane and plays a crucial role in cell cycle signaling, specifically in relation to apoptosis. PS exposure on the cell surface initiates blood clotting and facilitates the replication of various envelope viruses (Morizono and Chen, 2014). Recent studies have demonstrated that PS receptors enhance SARS-CoV-2 infection through virion-associated PS binding. In addition to host cells, a wide variety of gut microbes are also capable of synthesizing different types of PS (Vance and Tasseva, 2013). In addition, seven significantly downregulated metabolites, including dihydroneopterin triphosphate, dihydrobiopterin, pantetheine, DTMP, cortexolone, cortisol, and pregnenolone sulfate, were significantly associated with the depletion of *Bifidobacterium*, *Faecalibacterium,* and *Subdoligranulum*, as well as the over presence of *Fusobacterium* in feces. Reduction of dihydrotreterin and dihydrotreterin triphosphate can cause a deficiency of tetrahydrobiopterin (BH4), further resulting in impaired amino acid metabolism, and increased susceptibility to intestinal inflammation (Kinoshita et al., 2012). Metagenomic analysis has shown that a wide variety of gut microbes including *Bifidobacterium* and *Prevotella*, possess a BH4 biosynthesis pathway (Magnúsdóttir et al., 2015). Panthenine is involved in the biosynthesis of pantothenate (Vitamin B5) and CoA biosynthesis (Naquet et al., 2014). According to genomic analysis, some symbiotic bacteria in the gut, such as *Bacteroides fragilis*, *Prevotella copri* and some *Ruminococcus* spp. can produce pantetheine. The decrease of pantetheine can lead to the reduction in the production of coenzyme A, pantothenic acid, and cysteamine, which not only affect the metabolism of sugar, fat, protein, and the synthesis of some important substances such as neurotransmitters and steroid hormones but also cause the damage of intestinal immune barrier (Nitto and Onodera, 2013). Previous research based on metagenomic functional prediction has also shown that diarrheic calves had decreased abundances of genes responsible for folate biosynthesis, pantothenate and CoA biosynthesis (Gomez et al., 2017). The decrease in DTMP synthesis affects pyrimidine metabolism, blocking the biosynthesis of DNA and protein and increasing the susceptibility of the host to pathogenic microorganisms (Chon et al., 2017). Pregnenolone sulfate can be metabolized to produce cortexolone, cortisol, and aldosterone (Harteneck, 2013). Cortisol not only affects glucose metabolism but also plays an essential role in anti-inflammatory, anti-viral, anti-allergy and anti-shock (Almeida and Mendonca, 2020). Aldosterone is mainly responsible for regulating the metabolic balance of water and electrolytes. The reduction of pregnenolone sulfate, cortexolone, and cortisol in the intestinal tract of diarrheal calves infected with BCoV could lead to the disorder of energy, water, and electrolyte metabolism as well as a decline of immune function. It may aggravate diarrhea and increase the susceptibility of the animal to pathogenic microorganisms.

Increasing evidence suggests that SCFAs play an important role in maintaining gut and metabolic health (Blaak et al., 2020; He et al., 2020). Changes in the content of short-chain fatty acids in the intestine are associated with some diseases and intestinal inflammation in humans and animals (Parada Venegas et al., 2019). The effects of BRV and BCoV infection on intestinal SCFAs of newborn calves have not been documented in previous studies. However, in human and experimental animal models, it has also been found that the depletion of intestinal SCFAs accompanies virus infection in humans and animals. The fecal concentrations of SCFAs, including acetic acid, propionic acid, butyric acid, valerate, and caproic acid, were significantly lower in COVID-19 patients with severe illness than non-COVID-19 controls (Zhang et al., 2022). SARS-CoV-2 infection may be associated with the impaired capacity of the gut microbiome for SCFA production (Zhang et al., 2022). In this study, we observed that the concentrations of total SCFAs, acetic acid, propionic acid, and isocaproic acid in the BRV group as well as the concentration of propanoic acid in the BCoV group, were significantly lower than those of healthy calves. The loss of *Parabacteroides* and *Ruminococcus*, etc., was closely associated with the reduction of acetic acid, while the missing of *Parabacteroides*, *Ruminococcus*, *Fournierella*, and *Rikenellaceae_RC9_gut_group*, etc., were closely related to the decreased contents of isohexanoic acid. However, a significant reduction in propanoic acid in the feces of calves with BCoV-associated diarrhea was positively associated with the depletion of *Collinsella*. It is also reported that *Parabacteroides*, *Ruminococcus*, *Collinsella*, and other bacteria genera can produce SCFAs well in the anaerobic environment of the intestine (Li et al., 2018; Cui et al., 2022). Therefore, we speculate that the missing of these bacteria genera producing SCFA may contribute to the depletion of SCFA in the intestine of calves, which could promote intestinal inflammatory response, and exacerbate the imbalance of intestinal microbiota.

In the present study, we systematically analyzed the characteristics of fecal microbiota and metabolites in neonatal calves with diarrhea associated with BRV and BCoV infection, preliminarily elucidated the relationship among the infection of these two viruses, intestinal microbiota and metabolites, and laid a foundation for further revealing of the role of intestinal microbiota in neonatal calf diarrhea related to BRV and BCoV infection. However, there were some limitations in our study. First of all, fecal samples in this study were all from the same farm, and the research results could not adequately represent the alterations of intestinal microbiota in neonatal calves infected with the two viruses under different feeding conditions and management modes. Secondly, the intensity of viral infection, the stage of infection in the animals, and the severity of diarrhea may all have important effects on the results. Thirdly, there are many types of pathogens causing calf diarrhea. Although we detected the most common six diarrhea-related pathogens of newborn calves, the infection of other potential pathogens may affect the results. Finally, the microbial structure and function of different intestinal segments of calves are different. In this study, only rectal fecal samples were collected from calves, so it was not possible to accurately understand the change characteristics of the microbiota and metabolites in different intestinal sites of calves. In this study, although PICRUSt was used to predict microbiota functions based on 16S rRNA data, as most of the intestinal microbiota is still unknown or uncharacterized, the PICRUSt predictions are not accurate and comprehensive enough, and detailed analysis of microbial function based on metagenomics is still needed in the future. In future studies, we will further investigate the effects of BRV and BCoV infection on the function of intestinal microbiota, host immunity, and host metabolism based on a more representative experimental animal cohort and a more rigorous experimental protocol.

## 5. Conclusion

The diversity and composition of fecal microbiota were significantly changed in calves with diarrhea associated with BRV and BCoV infection. The significant increase of *Fusobacterium* and the decreased of bacteria genera, including *Faecalibacterium*, *Bifidobacterium*, *Erysipelatoclostridium*, *Parabacteroides*, *Collinsella*, and *Olsenella*, etc., were closely related to diarrhea associated with BRV and BCoV infection. Calves with diarrhea associated with BRV or BCoV infection had significantly changed in fecal metabolites. BRV infection-associated diarrhea group had a higher level of PC [16:1(9 Z)/16:1(9 Z)], LysoPE (0:0/22:0), LysoPC (P-16:0), LysoPE (0:0/18:0) and a lower concentration of dethiobiotin, which is closely correlated with Glycerophospholipid metabolism and Biotin metabolic pathway, respectively. There were two upregulated metabolites and 67 downregulated metabolites in BCoV infection-associated diarrhea group, which were closely correlated with the increase of harmful bacteria and the depletion of commensal bacteria. The downregulated metabolites were enriched in the pathways of Folate biosynthesis, Pantothenate and CoA biosynthesis, Pyrimidine metabolism, and Steroid hormone biosynthesis. The concentrations of some SCFA in BRV and BCoV groups were significantly lower than in healthy calves, which was associated with the depletion of SCFAs-producing bacteria.

## Data availability statement

The datasets presented in this study can be found in online repositories. The names of the repository/repositories and accession number(s) can be found at: https://www.ncbi.nlm.nih.gov/, PRJNA928849.

## Ethics statement

The animal study was reviewed and approved by Animal Experimental Ethical Review Committee of Ningxia University (Yinchuan, China; no. NXU-2019-051).

## Author contributions

SC, SG, and YY carried out the experimental design of this study. SC, QZ, and YY contributed to the experimental implementation and contributed to the article writing. SC contributed to the data analysis. SC, SG, YL, YM, and YY were involved in the sample collection and testing of this study. All authors contributed to the article and approved the submitted version.

## Funding

This research was supported by grants from Ningxia Hui Autonomous Region Key R&D Projects (nos. 2021BEF02028, 2021BEF01001, and 2020BBF03008) and Natural Science Foundation of Ningxia (nos. 2022AAC03231 and 2023AAC03051).

## Conflict of interest

The authors declare that the research was conducted in the absence of any commercial or financial relationships that could be construed as a potential conflict of interest.

## Publisher’s note

All claims expressed in this article are solely those of the authors and do not necessarily represent those of their affiliated organizations, or those of the publisher, the editors and the reviewers. Any product that may be evaluated in this article, or claim that may be made by its manufacturer, is not guaranteed or endorsed by the publisher.

## Footnotes

^1^ https://docs.scipy.org/doc/scipy/
