# Supplementary material for: Alterations of microbiota and metabolites in the feces of calves with diarrhea associated with rotavirus and coronavirus infections
Source: Front Microbiol. 2023 Aug 3;14:1159637. doi: 10.3389/fmicb.2023.1159637 (PMC10434556; doi:10.3389/fmicb.2023.1159637)
Supplement: Supplementary file 1 [file Data_Sheet_1.docx]

Supplementary Material

## Supplementary Figures

| 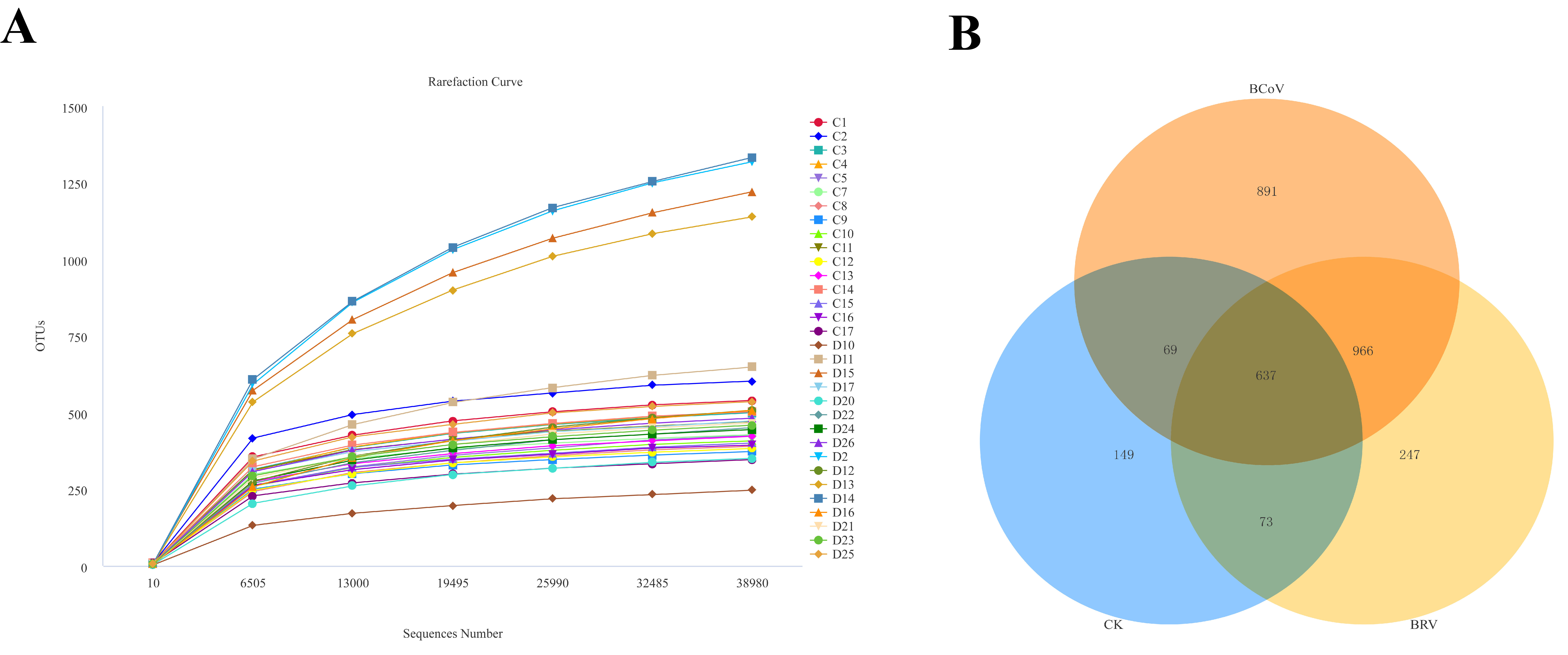 |
| --- |
| **Supplementary Figure 1.** The rarefaction curves and Venn map. (**A**)Rarefaction curves. The rarefaction curves of OTUs. (**B**)Venn map. The Venn map shows the numbers of OTUs (97% sequence identity). |

|  |
| --- |
| **Supplementary Figure 2.** Analysis of fecal microbiota composition in CK, BRV, and BCoV groups. (**A**)Bar chart of relative abundance at the phyla level for the CK and BRV groups. (**B**)Bar chart of relative abundance at the phyla level for the CK and BCoV groups. (**C**)Wilcoxon tests for phylum levels in the CK and BRV groups. (**D**)Wilcoxon tests for phylum levels in the CK and BCoV groups. (**E**)Bar chart of relative abundance at the genus level for the CK and BRV groups. (**F**)Bar chart of relative abundance at the genus level for the CK and BCoV groups. |

|  |
| --- |
| **Supplementary Figure 3.** Altered fecal metabolic profile in the CK, BRV, and BCoV groups. (**A**)CK and BRV group positive ion mode PCA diagram. (**B**)CK and BRV group negative ion mode PCA diagram. (**C**)CK and BCoV group positive ion mode PCA diagram. (**D**)CK and BCoV group negative ion mode PCA diagram. (**E**)CK and BRV group positive ion mode OPLS-DA diagram. (**F**)CK and BRV group negative ion mode OPLS-DA diagram. (**G**)CK and BCoV group positive ion mode OPLS-DA diagram. (**H**)CK and BCoV group negative ion mode OPLS-DA diagram. (**I**)CK and BRV group positive ion mode OPLS-DA permutation test. (**J**)CK and BRV group negative ion mode OPLS-DA permutation test. (**K**)CK and BCoV group positive ion mode OPLS-DA permutation test. (**L**)CK and BCoV group negative ion mode OPLS-DA permutation test. |

| 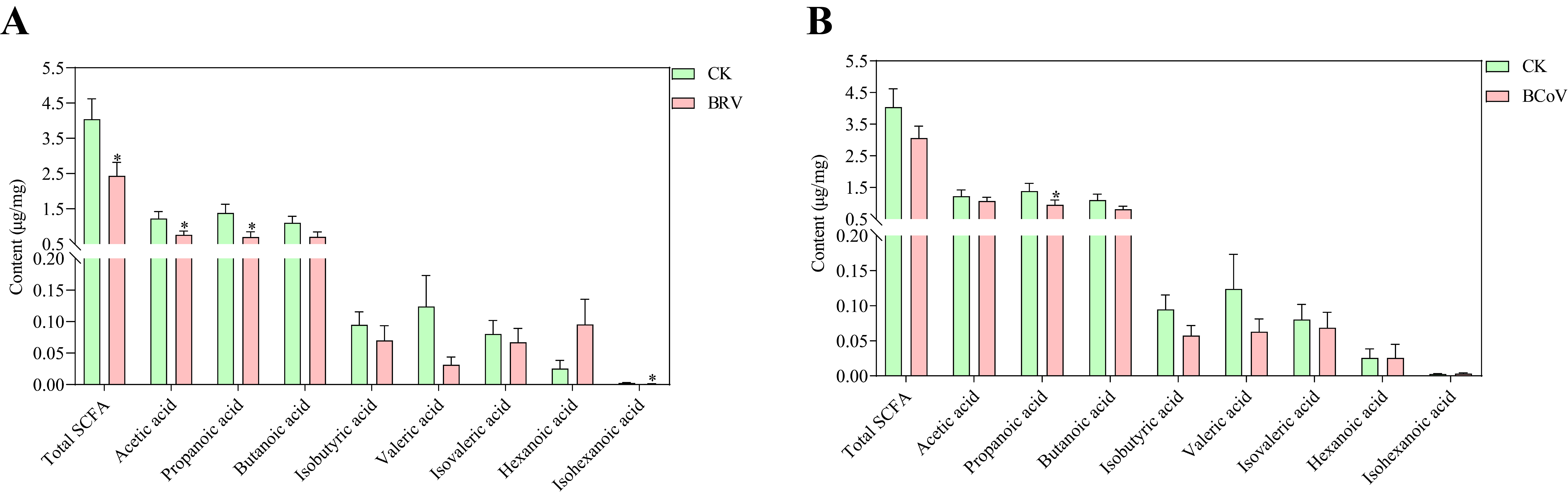 |
| --- |
| **Supplementary Figure 4.** Differences in fecal SCFAs content between CK, BRV, and BCoV groups. (**A**)Differences in fecal SCFAs content between CK and BRV groups. (**B**)Differences in fecal SCFAs content between CK and BCoV groups. *P<0.05. |

## **Supplementary Tables**

**Supplementary Table 1.** Information table of PCR primers for pathogen screening of calf diarrhea.

| **Pathogen** | **Targeted-location** | **Primer** | **Primer Sequence (5′-3′)** | **Size (bp)** | **Refere-nces** |
| --- | --- | --- | --- | --- | --- |
| BRV | VP6 gene | BRV-F | GACGGVGCRACTACATGGT | 380 | (Basera et al., 2010) |
|  |  | BRV-R | GTCCAATTCATNCCTGGTG |  |  |
| BCoV | N protein gene | BCoV-F | GCAATCCAGTAGTAGAGCGT | 730 | (Cho et al., 2001) |
|  |  | BCoV-R | CTTAGTGGCATCCTTGCCAA |  |  |
| BVDV | 5'UTR gene | BVDV-F | ATGCCCWTAGTAGGACTAGCA | 288 | (Choi et al., 2011) |
|  |  | BVDV-R | TCAACTCCATGTGCCATGTAC |  |  |
| *Cryptosporidium* spp. | SSU rRNA gene | SSU-F1 | TTCTAGAGCTAATACATGCG | 1320 | (Yin et al., 2013) |
|  |  | SSU-R1 | CCCATTTCCTTCGAAACAGGA |  |  |
|  |  | SSU-F2 | GGAAGGGTTGTATTTATTAGATAAAG | 840 |  |
|  |  | SSU-R2 | CTCATAAGGTGCTGAAGGAGTA |  |  |
| *Escherichia coli* K99 | fimbrial gene K99 (F5) | K99-F | TGGGACTACCAATGCTTCTG | 450 | (DebRoy et al., 2001) |
|  |  | K99-R | TATCCACCATTAGACGGAGC |  |  |
| BNoV | RdRp gene | BNoV-F | AGTTAYTTTTCCTTYTAYGGBGA | 532 | (Smiley et al., 2003) |
|  |  | BNoV-R | AGTGTCTCTGTCAGTCATCTTCAT |  |  |

**Supplementary Table 2.** Pathogen detection results.

| **Sample ID** | **Fecal score** | **BRV** | **BCoV** | **BNoV** | **BNoVBVDV** | ***Cryptosporidium* spp.** | ***Escherichia coli* K99** |
| --- | --- | --- | --- | --- | --- | --- | --- |
| W1 | 0 |  |  |  |  |  |  |
| W2 | 0 |  |  |  |  | + |  |
| W3 | 0 |  |  |  |  |  |  |
| W4 | 0 |  |  |  |  |  |  |
| W5 | 0 |  |  |  |  |  |  |
| W6 | 0 |  |  |  |  |  |  |
| W7 | 1 |  |  |  |  |  |  |
| W8 | 0 |  |  |  |  |  |  |
| W9 | 1 |  |  |  |  | + |  |
| P1 | 3 |  | + |  |  |  |  |
| P2 | 3 |  | + |  |  |  |  |
| P3 | 2 |  |  |  |  |  |  |
| P4 | 2 |  |  |  |  |  |  |
| P5 | 2 |  |  |  |  |  |  |
| P6 | 2 | + |  |  |  |  |  |
| P7 | 3 |  | + |  |  |  |  |
| P8 | 3 | + |  |  |  |  |  |
| P9 | 2 |  |  |  |  |  |  |
| P10 | 3 |  |  |  |  |  |  |
| P11 | 3 |  | + |  |  |  |  |
| P12 | 2 |  |  |  |  |  |  |
| P13 | 3 |  |  |  |  | + |  |
| P14 | 2 |  |  |  |  |  |  |
| P15 | 2 |  |  |  |  |  |  |
| P16 | 1 |  | + |  |  |  |  |
| a1 | 0 |  |  |  |  |  |  |
| a2 | 0 |  |  |  |  |  |  |
| a3 | 0 |  |  |  |  |  |  |
| a4 | 0 |  |  |  |  |  |  |
| a5 | 0 |  |  |  |  |  |  |
| a6 | 1 |  |  | + |  |  |  |
| a7 | 1 |  |  |  |  |  |  |
| a8 | 0 |  |  |  |  |  |  |
| a9 | 0 |  |  |  |  |  |  |
| a10 | 0 |  |  |  |  |  |  |
| a11 | 1 |  |  |  |  |  |  |
| a12 | 0 |  |  |  |  |  |  |
| a13 | 0 |  |  |  |  |  |  |
| a14 | 0 |  |  |  |  |  |  |
| a15 | 0 |  |  |  |  |  |  |
| b1 | 2 |  |  |  |  |  |  |
| b2 | 3 | + |  |  |  |  |  |
| b3 | 2 | + |  |  |  |  |  |
| b4 | 2 |  |  |  |  |  |  |
| b5 | 2 |  |  |  |  |  |  |
| b6 | 2 |  |  |  |  |  |  |
| b7 | 2 |  |  |  |  |  |  |
| b8 | 3 |  |  |  |  |  |  |
| b9 | 2 |  |  |  |  |  |  |
| b10 | 3 |  |  |  |  |  |  |
| b11 | 2 |  |  |  |  |  |  |
| b12 | 2 |  |  |  |  |  |  |
| b13 | 3 | + |  |  |  |  |  |
| b14 | 2 |  |  |  |  |  |  |
| b15 | 2 |  |  |  |  |  |  |
| k1 | 3 |  |  |  |  |  |  |
| k2 | 3 |  |  |  |  |  |  |
| k3 | 2 | + |  |  |  |  |  |
| k4 | 2 | + |  |  |  |  |  |
| k5 | 2 |  |  |  |  |  |  |
| k6 | 2 |  |  |  |  |  |  |
| k7 | 2 |  |  |  |  |  |  |
| k8 | 2 |  |  |  |  |  |  |
| k9 | 3 | + |  |  |  |  |  |
| k10 | 3 |  |  |  |  |  |  |
| k11 | 2 |  |  |  |  |  |  |
| k12 | 2 | + |  |  |  |  |  |
| k13 | 3 |  |  | + |  |  |  |
| k14 | 2 | + |  |  |  |  |  |
| J1 | 0 |  |  |  |  |  |  |
| J2 | 0 |  |  |  |  |  |  |
| J3 | 0 |  |  |  |  |  |  |
| J4 | 0 |  |  |  |  |  |  |
| J5 | 0 |  |  | + |  |  |  |
| J6 | 0 |  |  | + |  |  |  |
| E1 | 0 |  | + |  |  |  |  |
| E2 | 1 |  | + |  |  |  |  |
| E3 | 0 |  |  |  |  |  |  |
| E4 | 0 |  |  |  |  |  |  |
| E5 | 0 |  |  |  |  |  |  |
| E6 | 0 |  |  |  |  |  |  |
| E7 | 0 |  |  | + |  |  |  |
| E8 | 1 |  |  |  |  |  |  |
| E9 | 0 |  |  |  |  |  |  |
| E10 | 1 |  |  | + |  |  |  |
| F1 | 3 |  |  | + |  | + |  |
| F2 | 3 |  |  | + |  |  |  |
| F3 | 2 | + |  |  |  |  |  |
| F4 | 2 |  |  |  |  |  |  |
| F5 | 2 |  |  |  |  |  |  |
| F6 | 3 |  |  |  |  |  |  |
| F7 | 3 |  |  | + |  | + |  |
| F8 | 2 | + |  | + |  |  |  |
| F9 | 2 |  |  | + |  |  |  |
| X1 | 0 |  |  |  |  |  |  |
| X2 | 0 |  |  |  |  |  |  |
| X3 | 0 |  |  |  |  |  |  |
| X4 | 0 |  |  |  |  |  |  |
| X5 | 0 |  |  |  |  |  |  |
| X6 | 0 |  |  |  |  |  |  |
| X7 | 0 |  |  |  |  |  |  |
| X8 | 0 |  |  |  |  |  |  |
| X9 | 0 |  |  |  |  |  |  |
| X10 | 0 |  |  |  |  |  |  |
| X11 | 0 |  |  |  |  |  |  |
| Y1 | 2 |  |  |  |  |  |  |
| Y2 | 3 |  |  |  |  |  |  |
| Y3 | 2 |  |  |  |  |  |  |
| Y4 | 2 |  | + |  |  |  |  |
| Y5 | 3 |  |  | + |  |  |  |
| Y6 | 2 |  |  |  |  |  |  |
| Y7 | 3 | + |  |  |  |  |  |
| Y8 | 2 |  |  |  |  |  |  |
| Y9 | 3 | + |  |  |  | + |  |
| Y10 | 2 |  |  |  |  |  |  |
| Y11 | 3 |  |  |  |  |  |  |
| Y12 | 3 |  |  |  |  |  |  |
| C1 | 0 |  | + |  |  |  |  |
| C2 | 0 |  |  |  |  |  |  |
| C4 | 0 |  |  |  |  |  |  |
| C5 | 1 |  |  |  |  |  | + |
| C6 | 0 |  |  |  |  |  |  |
| C7 | 0 |  |  |  |  |  |  |
| C8 | 0 |  |  |  |  |  |  |
| C9 | 0 |  |  |  |  |  |  |
| C10 | 0 |  |  |  |  |  |  |
| C11 | 0 |  |  |  |  |  |  |
| D1 | 3 |  | + |  |  |  |  |
| D2 | 3 | + |  |  |  |  |  |
| D3 | 2 |  |  |  |  |  |  |
| D4 | 3 |  |  | + |  | + | + |
| D5 | 3 |  | + |  |  |  |  |
| D6 | 3 | + |  |  |  |  |  |
| D7 | 3 |  | + |  |  |  |  |
| D8 | 3 | + |  |  |  |  |  |
| D9 | 2 |  |  |  |  | + |  |
| D10 | 3 |  |  |  |  |  | + |
| L1 | 0 |  |  |  |  |  |  |
| L2 | 0 |  |  |  |  |  |  |
| L3 | 0 |  |  |  |  |  |  |
| L4 | 1 |  |  |  |  |  |  |
| L5 | 1 |  |  |  |  |  |  |
| L6 | 0 |  |  |  |  |  |  |
| L7 | 0 |  |  |  |  |  |  |
| L8 | 0 |  |  |  |  |  |  |
| L9 | 0 |  |  |  |  |  |  |
| M1 | 3 |  |  | + |  |  |  |
| M2 | 2 |  |  |  |  |  |  |
| M3 | 2 |  |  |  |  |  |  |
| M4 | 2 |  |  |  |  |  |  |
| M5 | 2 | + |  |  |  |  |  |
| M6 | 3 |  |  |  |  |  |  |
| M7 | 3 |  |  |  |  |  |  |
| M8 | 3 |  |  |  |  |  |  |
| M9 | 2 | + |  |  |  |  |  |
| M10 | 2 |  |  |  |  |  |  |
| M11 | 2 |  |  |  |  |  |  |
| M12 | 2 | + |  |  |  |  |  |
| M13 | 3 |  |  |  |  |  |  |
| M14 | 3 |  |  | + |  | + |  |
| H1 | 0 |  |  |  |  |  |  |
| H2 | 1 |  |  |  |  |  |  |
| H3 | 0 |  |  |  |  |  |  |
| H4 | 1 |  | + |  |  | + |  |
| H5 | 0 |  |  |  |  |  |  |
| H6 | 0 |  |  |  |  |  |  |
| H7 | 0 |  |  |  |  |  |  |
| H8 | 0 |  |  |  |  |  |  |
| H9 | 0 |  | + |  |  |  |  |
| H10 | 0 |  |  |  |  |  |  |
| I1 | 2 |  |  | + |  |  |  |
| I2 | 3 |  |  |  |  |  |  |
| I3 | 3 |  |  |  |  |  |  |
| I4 | 3 |  |  |  |  | + |  |
| I5 | 3 |  | + |  |  |  |  |
| I6 | 2 |  |  | + |  |  |  |
| I7 | 2 |  |  | + |  |  |  |
| I8 | 3 |  |  |  |  | + |  |
| I9 | 2 |  |  |  |  |  |  |
| I10 | 2 |  |  | + |  |  |  |
| A1 | 0 |  |  |  |  |  |  |
| A2 | 0 |  |  |  |  |  |  |
| A3 | 0 |  |  |  |  |  |  |
| A4 | 0 |  |  |  |  |  |  |
| A5 | 0 |  |  |  |  |  |  |
| A6 | 0 |  |  |  |  |  |  |
| A7 | 0 |  |  |  |  |  |  |
| A8 | 0 |  |  |  |  |  |  |
| B2 | 2 |  |  |  |  |  |  |
| B3 | 2 |  |  |  |  |  |  |
| B4 | 2 |  |  |  |  |  |  |
| B5 | 3 |  |  |  |  |  |  |
| B6 | 2 |  |  |  |  |  |  |
| B7 | 3 |  |  |  |  |  |  |
| B8 | 3 |  |  |  |  |  |  |

Note: The fecal score of 0,1 is healthy and normal, and 2,3 is diarrhea. "**+**" means positive, and no mark means negative.

Based on the fecal score and pathogen detection results, samples with a fecal score of 0 and pathogen detection of negative samples were classified as group CK (Sample ID: W1, W3, W4, W5, W6, W8, H1, H3, H5, H6, H7, H8, H10, E5, E6, E9). Samples with a fecal score of 3 and only detected BRV were classified as group BRV (Sample ID: b13, k9, P8, Y7, b2, D2, D6, D8), while samples with a fecal score of 3 and only detected BCoV were classified as group BCoV (Sample ID: I5, P1, P2, P7, P11, D1, D5, D7). A total of 32 samples were sequenced by 16S rRNA amplicon. Afterward, eight samples were selected from the CK group (Sample ID: W3, W4, W5, W6, H1, H3, H7, H10), six samples were selected from the BRV group (Sample ID: b13, k9, b2, D2, D6, D8), and six samples were selected from the BCoV group (Sample ID: P1, P2, P11, D1, D5, D7) for metabolome sequencing analysis.

**Supplementary Table 3.** Sample sequence statistics table.

| **Sample ID** | **16S Sample_ID** | **Effective_Tag** | **Taxon_Tag** | **Unclassified_Tag** | **Unique_Tag** | **OTU_num** |
| --- | --- | --- | --- | --- | --- | --- |
| I5 | D2 | 60591 | 57324 | 20 | 3247 | 1488 |
| b13 | D10 | 64817 | 63683 | 46 | 1088 | 296 |
| k9 | D11 | 60748 | 54733 | 2 | 6013 | 721 |
| P1 | D12 | 61444 | 59685 | 0 | 1759 | 573 |
| P2 | D13 | 56246 | 53296 | 13 | 2937 | 1334 |
| P7 | D14 | 65190 | 61587 | 3 | 3600 | 1508 |
| P8 | D15 | 41958 | 38968 | 15 | 2975 | 1221 |
| P11 | D16 | 63996 | 62573 | 6 | 1417 | 578 |
| Y7 | D17 | 65692 | 62321 | 87 | 3284 | 510 |
| b2 | D20 | 66346 | 65185 | 0 | 1161 | 385 |
| D1 | D21 | 66171 | 64201 | 11 | 1959 | 523 |
| D2 | D22 | 67205 | 65614 | 1 | 1590 | 496 |
| D5 | D23 | 65747 | 63352 | 0 | 2395 | 508 |
| D6 | D24 | 65239 | 63683 | 1 | 1555 | 504 |
| D7 | D25 | 66417 | 64115 | 8 | 2294 | 595 |
| D8 | D26 | 65815 | 63252 | 0 | 2563 | 518 |
| W1 | C1 | 60300 | 57492 | 1 | 2807 | 581 |
| W3 | C2 | 64300 | 60335 | 0 | 3965 | 634 |
| W4 | C3 | 64769 | 61677 | 0 | 3092 | 543 |
| W5 | C4 | 65754 | 63144 | 3 | 2607 | 560 |
| W6 | C5 | 61351 | 57547 | 46 | 3758 | 480 |
| W8 | C7 | 67111 | 64189 | 6 | 2916 | 480 |
| H1 | C8 | 69007 | 67095 | 9 | 1903 | 433 |
| H3 | C9 | 66673 | 64347 | 4 | 2322 | 414 |
| H5 | C10 | 64129 | 62475 | 0 | 1654 | 440 |
| H6 | C11 | 63271 | 60135 | 48 | 3088 | 507 |
| H7 | C12 | 69333 | 67573 | 2 | 1758 | 417 |
| H8 | C13 | 61768 | 60109 | 1 | 1658 | 471 |
| H10 | C14 | 63896 | 62112 | 10 | 1774 | 546 |
| E5 | C15 | 67959 | 65797 | 4 | 2158 | 429 |
| E6 | C16 | 60747 | 58769 | 4 | 1974 | 433 |
| E9 | C17 | 68717 | 66717 | 15 | 1985 | 379 |

**Supplementary Table 4.** Different metabolites of the CK group and BRV group.

| **Metabolite** | **Regulate** | **VIP** | **FC** | **P_value** | **FDR** |
| --- | --- | --- | --- | --- | --- |
| 5-(2-Methylpropyl)tetrahydro-2-oxo-3-furancarboxylic acid | down | 2.288 | 0.4449 | 0.0001423 | 0.00198 |
| (R)-8-Acetoxycarvotanacetone | down | 2.0519 | 0.2083 | 0.005611 | 0.01965 |
| MG(0:0/20:5(5Z,8Z,11Z,14Z,17Z)/0:0) | down | 2.0315 | 0.3143 | 0.007927 | 0.02482 |
| 2-hydroxy-3,4-diphenylpentanedioic acid | down | 2.763 | 0.1727 | 0.0009984 | 0.006351 |
| Caffeoylferuloylspermidine | down | 2.0946 | 0.5623 | 0.0001073 | 0.001678 |
| Auberganol | down | 2.3412 | 0.4539 | 0.0001009 | 0.001622 |
| LysoPE(0:0/18:0) | up | 2.1297 | 1.6093 | 0.0001742 | 0.002234 |
| LysoPC(P-16:0) | up | 2.2745 | 2.0828 | 0.006771 | 0.02225 |
| LysoPE(0:0/22:0) | up | 2.7416 | 2.6347 | 0.0000003563 | 0.00006472 |
| Polyporusterone B | down | 2.9923 | 0.1665 | 0.0000001281 | 0.00004329 |
| Methyl glucosinolate | down | 2.1394 | 0.5308 | 0.0003275 | 0.003218 |
| 3-Oxo-1,4,11(13)-eudesmatrien-12-oic acid | down | 2.5889 | 0.1729 | 0.00001616 | 0.0005593 |
| 2-Hydroxyenterodiol | down | 2.1035 | 0.2783 | 0.004744 | 0.01747 |
| 2-(hydroxymethyl)-2-methyl-2H-chromen-5-ol | down | 2.0453 | 0.2983 | 0.01536 | 0.03994 |
| 1-Methoxy-3-(4-hydroxyphenyl)-2E-propenal 4'-glucoside | down | 2.3911 | 0.3451 | 0.00529 | 0.01719 |
| 5'-Hydroxy-3',4',7-trimethoxyflavan | down | 2.1213 | 0.3402 | 0.001868 | 0.008299 |
| Asparaginyl-Tryptophan | down | 2.0602 | 0.5493 | 0.0001672 | 0.001749 |
| PC(16:1(9Z)/16:1(9Z)) | up | 2.4562 | 2.4322 | 0.00001153 | 0.0003468 |
| Momordenol | down | 2.006 | 0.5141 | 0.003207 | 0.01208 |
| 6,15-diketo-13,14-dihydro Prostaglandin F1alpha | down | 2.6265 | 0.2319 | 0.0002867 | 0.002416 |
| Cinncassiol C3 | down | 2.0088 | 0.6259 | 0.0002357 | 0.002139 |
| L-Hexanoylcarnitine | down | 2.038 | 0.6142 | 0.00049 | 0.003402 |
| N-trans-p-Coumaroyloctopamine | down | 2.0026 | 0.4485 | 0.00006934 | 0.001053 |
| 12-Hydroxy-7-oxo-8,11,13-abietatrien-18-al | down | 2.1138 | 0.5674 | 0.00001288 | 0.0003683 |
| Discadenine | down | 2.2053 | 0.4306 | 0.007284 | 0.0217 |
| Dethiobiotin | down | 2.4247 | 0.0069 | 0.0002837 | 0.002396 |

Note: Monoacylglyceride (MG), Lysophosphatidylethanolamine (LysoPE), Lysophosphatidylcholine (LysoPC), Phosphatidylcholine (PC)

**Supplementary Table 5.** Different metabolites of the CK group and BCoV group.

| **Metabolite** | **Regulate** | **VIP** | **FC** | **P_value** | **FDR** |
| --- | --- | --- | --- | --- | --- |
| Methyl (3b,11x)-3-Hydroxy-8-oxo-6-eremophilen-12-oate | down | 2.0006 | 0.7521 | 0.01757 | 0.1545 |
| Dihydrobiopterin | down | 2.4938 | 0.504 | 0.02521 | 0.1743 |
| 2'-Oxoaloesol 7-glucoside | down | 2.78 | 0.7009 | 0.0008296 | 0.07456 |
| Ethyl beta-D-glucopyranoside | down | 2.6499 | 0.5993 | 0.00002116 | 0.0331 |
| Isopropyl apiosylglucoside | down | 2.1604 | 0.7234 | 0.001051 | 0.07846 |
| 3,4-Dimethyl-5-propyl-2-furanheptanoic acid | down | 2.3944 | 0.6208 | 0.02296 | 0.1671 |
| Pregnenolone sulfate | down | 2.1782 | 0.6953 | 0.001148 | 0.07864 |
| Prehumulinic acid | down | 2.0074 | 0.7544 | 0.01969 | 0.1613 |
| Gibberellin A3 | down | 2.193 | 0.771 | 0.00998 | 0.1364 |
| 3-Oxo-4,6-choladienoic acid | down | 2.0168 | 0.8077 | 0.008033 | 0.1299 |
| Indole-3-methyl acetate | down | 2.0936 | 0.7235 | 0.002417 | 0.09541 |
| Tryptophyl-Glutamine | down | 2.0082 | 0.7986 | 0.01199 | 0.1394 |
| FA(18:4(OH2)) | down | 2.7393 | 0.5339 | 0.01513 | 0.1504 |
| 7-hydroxygranisetron | down | 2.347 | 0.7006 | 0.005752 | 0.1195 |
| Caffeoylferuloylspermidine | down | 2.0173 | 0.732 | 0.03546 | 0.1996 |
| Polyporusterone B | down | 2.6745 | 0.4928 | 0.007055 | 0.1249 |
| Cortisol  皮质醇 | down | 2.0996 | 0.7384 | 0.01982 | 0.1613 |
| Isoachifolidiene | down | 2.0351 | 0.8323 | 0.008687 | 0.1333 |
| Diplodiatoxin | down | 2.0197 | 0.7589 | 0.03525 | 0.1993 |
| 5-Megastigmen-7-yne-3,9-diol 3-glucoside | down | 2.4032 | 0.6786 | 0.003325 | 0.1056 |
| Methyl glucosinolate | down | 2.0106 | 0.7192 | 0.01486 | 0.1503 |
| Hydroxypelenolide | down | 2.1195 | 0.6617 | 0.04349 | 0.2182 |
| Dihydroneopterin triphosphate | down | 2.365 | 0.6508 | 0.008074 | 0.1303 |
| NPC | down | 2.4184 | 0.7266 | 0.003833 | 0.11 |
| Auxin b | down | 2.1001 | 0.8189 | 0.007446 | 0.1276 |
| Diosbulbin F | down | 2.059 | 0.6614 | 0.01227 | 0.1406 |
| Homoeriodictyol 4'-isobutyrate | down | 2.2785 | 0.6773 | 0.00184 | 0.09101 |
| Cortexolone | down | 2.5914 | 0.5462 | 0.006965 | 0.1249 |
| Ciceritol | down | 2.0791 | 0.7335 | 0.003844 | 0.11 |
| Alectrol | down | 2.1091 | 0.7642 | 0.03443 | 0.1976 |
| Dioscoretine | down | 2.2468 | 0.6993 | 0.00218 | 0.09248 |
| Dityrosine | down | 2.06 | 0.7537 | 0.01336 | 0.1443 |
| DTMP | down | 2.5055 | 0.712 | 0.0002748 | 0.06263 |
| 3-(4-Isopropylphenyl)propanal | down | 2.2649 | 0.7718 | 0.007554 | 0.1277 |
| O-Desmethyl-lacosamide | down | 2.0197 | 0.7608 | 0.01093 | 0.1376 |
| Penicilloic acid | down | 2.4831 | 0.6768 | 0.01672 | 0.1529 |
| Avenic acid B | down | 2.1891 | 0.6398 | 0.04975 | 0.2242 |
| 16b-Hydroxyestradiol | down | 2.0771 | 0.7623 | 0.0005197 | 0.1023 |
| N-(1-Deoxy-1-fructosyl)phenylalanine | down | 2.1939 | 0.7497 | 0.001662 | 0.1095 |
| Desmethylofloxacin | down | 2.5516 | 0.6402 | 0.0005103 | 0.1023 |
| Desmethyl fluvoxamine | down | 2.138 | 0.7399 | 0.01364 | 0.1444 |
| Oxoproflaxacin | down | 2.8089 | 0.5662 | 0.002554 | 0.1122 |
| 5-Hydroxyindoleacetaldehyde | down | 2.147 | 0.7181 | 0.006238 | 0.1264 |
| Fusarochromanone | down | 2.5972 | 0.4893 | 0.0169 | 0.1532 |
| (R)-1-O-[b-D-Apiofuranosyl-(1->2)-b-D-glucopyranoside]-1,3-octanediol | up | 3.1903 | 1.9753 | 0.001533 | 0.1095 |
| PS(DiMe(11,3)/DiMe(9,5)) | up | 2.2393 | 1.4773 | 0.008318 | 0.1277 |
| O-Desmethylvenlafaxine glucuronide | down | 2.358 | 0.6541 | 0.0002918 | 0.1023 |
| Glabrin D | down | 2.134 | 0.7922 | 0.001878 | 0.1095 |
| Alpha-Trisaccharide | down | 2.2751 | 0.7553 | 0.006712 | 0.1277 |
| Sarmentosin | down | 2.1554 | 0.682 | 0.009587 | 0.1326 |
| Niazimicin A | down | 2.1832 | 0.7404 | 0.00797 | 0.1277 |
| Cinncassiol C3 | down | 2.2206 | 0.7548 | 0.01255 | 0.141 |
| Tryptophan (Trp) derivative | down | 2.2437 | 0.7437 | 0.006376 | 0.1265 |
| Tyrosyl-Isoleucine | down | 2.0948 | 0.7843 | 0.00437 | 0.1185 |
| Pantetheine | down | 2.1719 | 0.7364 | 0.00004512 | 0.07241 |
| 5'-S-Methylthioadenosine | down | 2.2963 | 0.722 | 0.0005574 | 0.1023 |
| [(5-hydroxy-2-methyl-2H-chromen-2-yl)methoxy]sulfonic acid | down | 2.4698 | 0.6236 | 0.002546 | 0.1122 |
| Phenylalanyl-Tryptophan | down | 2.1519 | 0.7421 | 0.001597 | 0.1095 |
| L-Hexanoylcarnitine | down | 2.1018 | 0.7559 | 0.02513 | 0.1732 |
| N-trans-p-Coumaroyloctopamine | down | 2.0846 | 0.6217 | 0.03351 | 0.1897 |
| Fenirofibrate | down | 2.4671 | 0.5475 | 0.02074 | 0.1625 |
| Undecanedioic acid | down | 2.0918 | 0.7689 | 0.01696 | 0.1532 |
| 4-Oxo-norfloxacin | down | 2.6435 | 0.6485 | 0.001358 | 0.1095 |
| Menthyl pyrrolidone carboxylate | down | 2.3416 | 0.7491 | 0.001187 | 0.1088 |
| Ile-lle-OH | down | 2.0137 | 0.7924 | 0.009237 | 0.1319 |
| 3-Hydroxydodecanedioic acid | down | 2.2068 | 0.7706 | 0.008318 | 0.1277 |
| 3-hydroxyhexanoyl carnitine | down | 2.1558 | 0.7508 | 0.004634 | 0.1185 |
| Armillane | down | 2.2197 | 0.7593 | 0.00677 | 0.1277 |
| Glutaminyltyrosine | down | 2.2455 | 0.6267 | 0.007483 | 0.1277 |

Note: Fatty acid (FA), Deoxythymidylic acid (DTMP), Phosphatidylserine (PS).

**References:**

Basera, S. S., Singh, R., Vaid, N., Sharma, K., Chakravarti, S., and Malik, Y. P. S. (2010). Detection of rotavirus infection in bovine calves by RNA-PAGE and RT-PCR. *Indian Journal of Virology*. 21, 144-147. doi: 10.1007/s13337-010-0017-9

Cho, K. O., Hasoksuz, M., Nielsen, P. R., Chang, K. O., Lathrop, S., and Saif, L. J. (2001). Cross-protection studies between respiratory and calf diarrhea and winter dysentery coronavirus strains in calves and RT-PCR and nested PCR for their detection. *Arch. Virol.* 146, 2401-2419. doi: 10.1007/s007050170011

Choi, K., and Song, M. (2011). Epidemiological observations of bovine viral diarrhea virus in Korean indigenous calves. *Virus Genes*. 42, 64-70. doi: 10.1007/s11262-010-0542-z

DebRoy, C., and Maddox, C. W. (2001). Identification of virulence attributes of gastrointestinal Escherichia coli isolates of veterinary significance. *Animal Health Research Reviews*. 2, 129-140. doi: 10.1079/AHRR200131

Smiley, J. R., Hoet, A. E., Traven, M., Tsunemitsu, H., and Saif, L. J. (2003). Reverse transcription-PCR assays for detection of bovine enteric caliciviruses (BEC) and analysis of the genetic relationships among BEC and human caliciviruses. *J. Clin. Microbiol.* 41, 3089-3099. doi: 10.1128/JCM.41.7.3089-3099.2003

Yin, J., Yuan, Z., Shen, Y., Zhang, J., Jiang, Y., and Cao, J. (2013). Molecular identification of Cryptosporidium spp. From animal sources in China. *J. Infect. Dev. Countr.* 7, 1020-1022. doi: 10.3855/jidc.3540
